# Near-ideal van der Waals rectifiers based on all-two-dimensional Schottky junctions

**DOI:** 10.1038/s41467-021-21861-6

**Published:** 2021-03-09

**Authors:** Xiankun Zhang, Baishan Liu, Li Gao, Huihui Yu, Xiaozhi Liu, Junli Du, Jiankun Xiao, Yihe Liu, Lin Gu, Qingliang Liao, Zhuo Kang, Zheng Zhang, Yue Zhang

**Affiliations:** 1 Beijing Advanced Innovation Center for Materials Genome Engineering, Beijing Key Laboratory for Advanced Energy Materials and Technologies, University of Science and Technology Beijing, Beijing, People’s Republic of China; 2 State Key Laboratory for Advanced Metals and Materials, School of Materials Science and Engineering, University of Science and Technology Beijing, Beijing, People’s Republic of China; 3 Collaborative Innovation Center of Quantum Matter, Beijing, China; 4 Beijing National Laboratory for Condensed Matter Physics, Institute of Physics, Chinese Academy of Sciences, Beijing, China

**Keywords:** Two-dimensional materials, Electronic devices

## Abstract

The applications of any two-dimensional (2D) semiconductor devices cannot bypass the control of metal-semiconductor interfaces, which can be severely affected by complex Fermi pinning effects and defect states. Here, we report a near-ideal rectifier in the all-2D Schottky junctions composed of the 2D metal 1 T′-MoTe_2_ and the semiconducting monolayer MoS_2_. We show that the van der Waals integration of the two 2D materials can efficiently address the severe Fermi pinning effect generated by conventional metals, leading to increased Schottky barrier height. Furthermore, by healing original atom-vacancies and reducing the intrinsic defect doping in MoS_2_, the Schottky barrier width can be effectively enlarged by 59%. The 1 T′-MoTe_2_/healed-MoS_2_ rectifier exhibits a near-unity ideality factor of ~1.6, a rectifying ratio of >5 × 10^5^, and high external quantum efficiency exceeding 20%. Finally, we generalize the barrier optimization strategy to other Schottky junctions, defining an alternative solution to enhance the performance of 2D-material-based electronic devices.

## Abstract (web summary)

Here, a defect healing method is used to tune the height and width of the Schottky barrier at the interface between 2D metals and 2D semiconductors, leading to the realization of van der Waals rectifiers with enhanced performance.

## Introduction

**Fig. 1:**
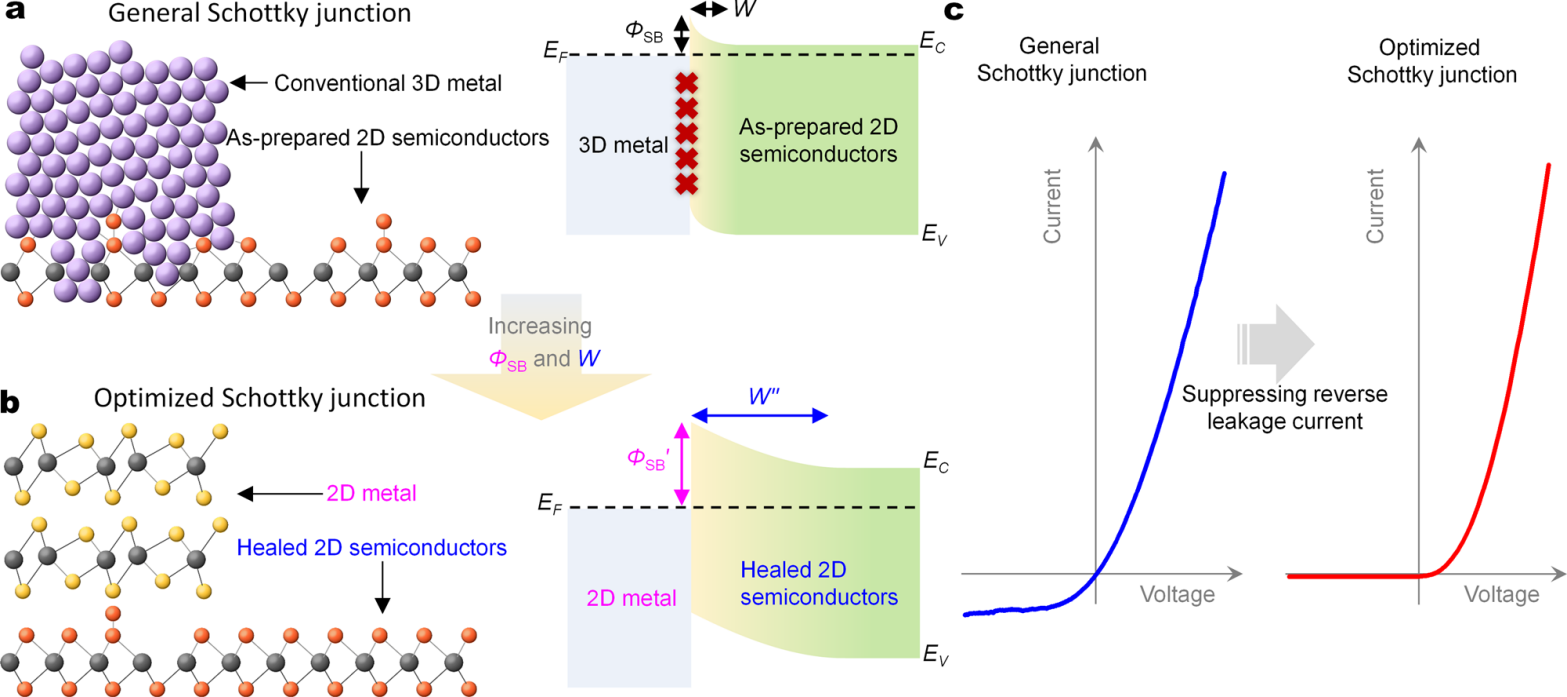
Design and novelties of all 2D Schottky junctions for ideal rectifiers. **a**, **b** Schematic diagrams of atomic structure (left) and band structure (right) of general (**a**) and optimized (**b**) Schottky junctions. The purple sphere is the element of the conventional metal electrode, and the yellow, orange, and gray spheres are the composed atoms of 2D metals and semiconductors, respectively. The red cross suggests the Fermi pinning effect. The yellow gradient areas refer to the depletion region of Schottky junction. Both the lattice defects involved by the evaporation of conventional two-dimensional (3D) metals, the decaying metallic wavefunction of conventional metals, and the interface states will cause the severe Fermi pinning effect which results in the small Schottky barrier height ${{\varPhi}} _{\mathrm{SB}}$. On the other hand, the intrinsic defect doping in as-prepared 2D semiconductors will render the high carrier concentration, which brings about the tiny Schottky barrier width *W*. The small height ${{\varPhi}} _{\mathrm{SB}}$ and tiny width *W* of Schottky barrier together will lead to the large reverse leakage current of the Schottky diode. **c** Current–voltage curves of general (left) and optimized (right) Schottky junctions.

Metal-semiconductor interfaces are crucial to the operations and applications of every semiconductor devices^1^, and are at the basis of Schottky diodes. With excellent on–off switching characteristics, Schottky diodes often replace p–n diodes in many essential components of modern electronics and optoelectronics^2–5^. Recently, without the constraint limits of lattice matching and processing compatibility requirements of conventional bonded heterojunctions, two-dimensional (2D) van der Waals (vdWs) heterojunctions have attracted widespread interests^6–10^. To date, the traditional merits of Schottky diodes are not reproduced in 2D material-based Schottky diodes but are replaced by many deficiencies, such as excessive reverse leakage current (i.e., large power consumption), low current rectifying ratio, and poor ideality factor^4,11–15^. This is attributed to the composition effect of the low Schottky barrier height and the tiny Schottky barrier width in 2D Schottky diodes in Fig. 1a, which are originated respectively from the strong Fermi pinning effect of the metal–semiconductor junctions and the ultrathin body thickness^2,5,16–20^.

Both achieving the designed Schottky barrier height and precisely controlling the barrier width are crucial for realizing the full potential of Schottky diodes. For Schottky barrier height, transferring metals or employing 2D metals can build weak Fermi pinned Schottky junctions to roughly satisfy the personalized barrier height^2,21–23^. The 2D metals (e.g., 1T′-MoTe_2_, 1T′-WTe_2_, 1T′-PtSe_2_, and 2H-NbSe_2_), by contrast, have more potential in creating high-grade Schottky junctions because their Schottky barrier heights strictly follow the trend of the Schottky–Mott model. Since 2D metals can form the weaker chemical bonding interface with 2D vdWs semiconductors and efficiently suppress the metal-induced gap states in conventional Schottky junctions^22,24,25^. The metal-induced gap states are one of the important sources of the Fermi pinning effect^2^.

On the other hand, the tunneling currents widely exist in the 2D semiconductor-based Schottky junctions^18^, which is highly related to its tiny Schottky barrier widths and will form the large leakage currents to cause the high power consumptions of diodes. To date, the adjustments of the Schottky barrier widths often rely on surface absorption chemical doping, which is environment unstable and inefficient in controlling the electronic structures^26,27^. Lattice defect healing is an optimal strategy to efficiently and stably decrease the intrinsic defect doping of ultrathin 2D materials^28,29^.

Here, we report a near-ideal Schottky diode in 1T′-MoTe_2_/MoS_2_ Schottky junction composed of the metallic 1T′-MoTe_2_ and the semiconducting monolayer MoS_2_ in Fig. 1b. We show constructing all 2D Schottky junctions can greatly reduce the Fermi pinning effect in the metal–semiconductor interfaces. Furthermore, we firstly demonstrate that by healing original defects, shrinking the intrinsic defect doping in monolayer MoS_2_ can effectively enlarge the Schottky barrier width to significantly suppress the reverse tunneling leakage current in Fig. 1c. After the defect healing in monolayer MoS_2_, the 1T′-MoTe_2_/healed-MoS_2_ Schottky diode shows a near-unity ideality factor of ~1.6, a high rectifying ratio of >5 × 10^5^ in monolayer semiconductor-based Schottky diodes to date, and high external quantum efficiency of >20%. The Schottky barrier optimization strategy is generally applicable to other Schottky junctions, including Pd/MoS_2_, 1T′-MoTe_2_/WS_2_, 1T-PtSe_2_/MoS_2_, and 1T-PtSe_2_/WS_2_. This work provides an alternative solution for improving the performance of 2D material-based Schottky junctions.

## Results

### Basic characterizations of the Schottky junctions

**Fig. 2:**
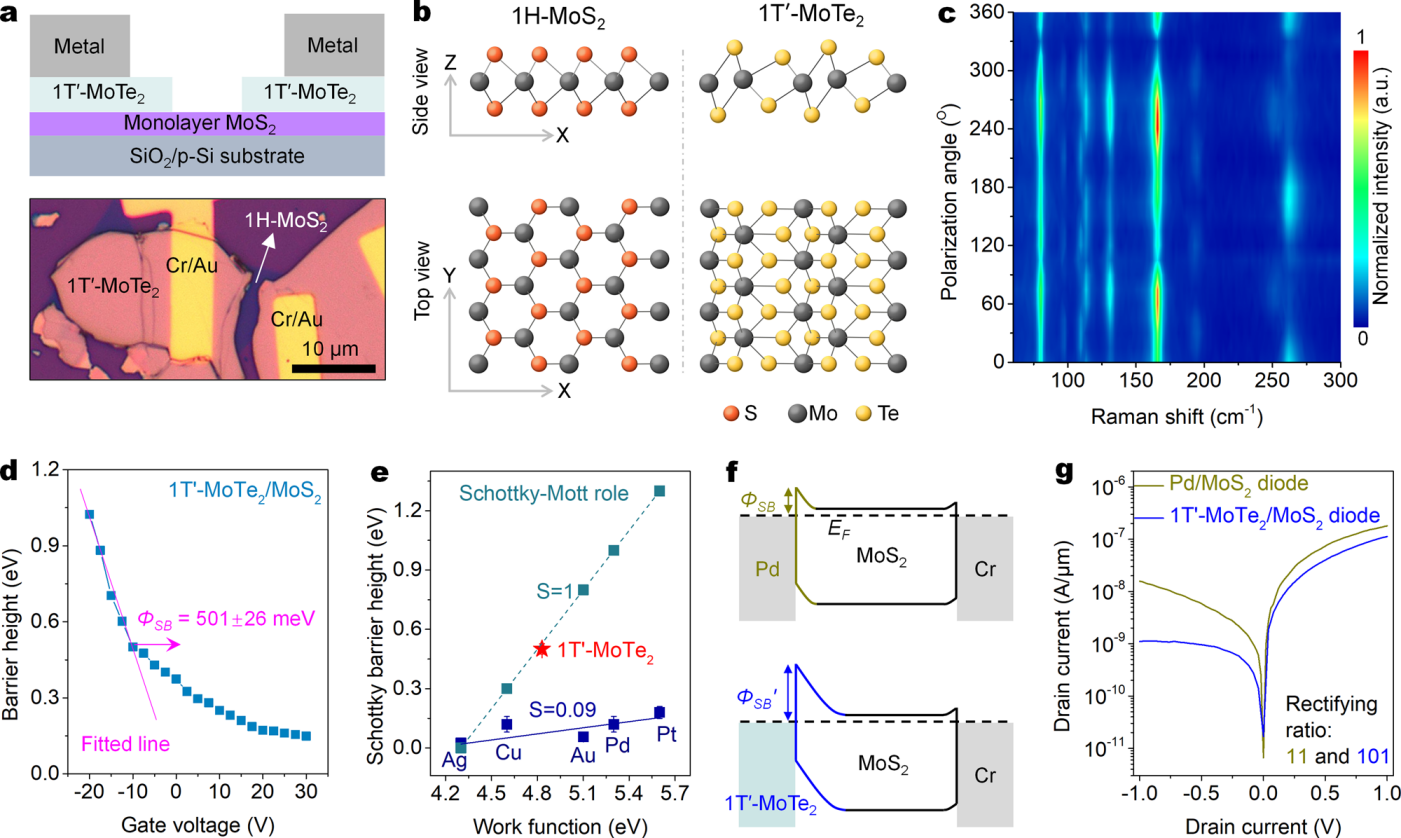
The 1T′-MoTe_2_/MoS_2_ Schottky junction with large Schottky barrier height and weak Fermi pinning effect. **a** Schematic diagram (top) and optical micrograph (bottom) of a monolayer MoS_2_ FET with symmetric 1T′-MoTe_2_ electrodes. **b** Crystal structures of the 1H-MoS_2_ (left) and 1T′-MoTe_2_ (right) monolayers. **c** Angle-resolved Raman spectra of the mechanically exfoliated 1T′-MoTe_2_ flake. **d** Barrier heights of the 1T′-MoTe_2_/MoS_2_ Schottky junction as a function of gate voltage obtained from Supplementary Fig. 4. The Schottky barrier height is extracted under a flat band gate voltage condition, which is responsible for the start of deviations from the linear behavior. **e** Comparisons of the extracted Schottky barrier height (SBH) between monolayer MoS_2_ and the electrodes of evaporated 3D metals (extracted from ref. ^2^) and the 2D metal 1T′-MoTe_2_. The gunmetal dash line and points are the calculated SBH from the ideal Schottky–Mott model. The blue solid line is fitted line of experimental data. **f**, **g** Band diagrams (**f**) and output curves (**g**) of the monolayer MoS_2_ diodes with the Pd and 1T′-MoTe_2_ electrodes. All gate voltages are 0 V.

The idea and strategy of the Schottky junction optimization are shown in Fig. 1a–c. The detailed differences between the general and optimized Schottky junctions by the conventional and 2D metals are summarized in Fig. 1a, b. A monolayer MoS_2_ transistor with symmetric 1T′-MoTe_2_ electrodes in Fig. 2a was built to characterize the Schottky barrier height between monolayer MoS_2_ and 1T′-MoTe_2_. The monolayer MoS_2_ and few-layer 1T′-MoTe_2_ are chemical vapor deposition (CVD) grown and mechanically exfoliated (‘Methods’), respectively. The 1H-MoS_2_ has a trigonal prismatic structure and lacks inversion symmetry; while the 1T′-MoTe_2_ has a distorted octahedral structure and inversion symmetry in Fig. 2b. The intensities of the several modes (e.g., 194 cm^−1^) exhibit a four-lobed shape with four maximum-intensity angles at ~10°, ~100°, ~190°, and ~280° in Fig. 2c and Supplementary Fig. 1. This data conform to the polarization dependence of the Raman signals of 1T′-MoTe_2_ films^30^. Besides, none of the relevant Raman spectra about 2H-MoTe_2_ (*A*_1*g*_/*E*_2*g*_: 170/231 cm^−1^) are observed (Supplementary Fig. 2b), indicating that the material fabrication process did not induce a 1T′-to-2H phase transition^12,31^.

### Characterization of Schottky barrier height

Kelvin probe force microscopy (KPFM) was used to characterize the work function of the metallic 1T′-MoTe_2_ nanosheet as ~4.83 eV (Supplementary Fig. 3c, d). The Schottky barrier height is measured as ~501 ± 26 meV in Fig. 2d by the variable temperature study (Supplementary Fig. 4), and is the same as the largest Schottky barrier height of 0.5 eV in the reported all 2D Schottky junctions^32^. In the Schottky–Mott rule, the Schottky barrier height in the Schottky junction is defined as^2,3^:

1 $${{\varPhi}} _{\mathrm{SB}} = {{\varPhi}} _M - \chi _S$$

where ${{\varPhi}} _M$ and ${\it{{\chi}}} _S$ are the work function of the metal electrode and the electron affinity of the n-type semiconductors, respectively. The $\chi _S$ of monolayer MoS_2_ previously reported is ~4.3 eV (ref. ^28,32^), implying the theoretical Schottky barrier height ${{\varPhi}} _{\mathrm{SB}}$ of the 1T′-MoTe_2_/MoS_2_ Schottky junction should be ~0.53 eV. The experimental and theoretical values are close, suggesting that our constructed 1T′-MoTe_2_/MoS_2_ Schottky junctions are excellent and largely follow the Schottky–Mott rule.

The work function of Pd is ~5.3 eV (ref. ^2^), which is larger than that of ~4.83 eV of 1T′-MoTe_2_. It should be noted that this work function of 1T′-MoTe_2_ was measured under the atmosphere environment and may be slightly different from the true vacuum value. According to the Schottky–Mott rule, the Pd/MoS_2_ Schottky junction should possess a larger Schottky barrier height than the 1T′-MoTe_2_/MoS_2_ Schottky junction. However, the Schottky barrier height of the 1T′-MoTe_2_/MoS_2_ Schottky junction is significantly higher than the reported values of ~0.12 eV of the Pd/MoS_2_ Schottky junction in Fig. 2e (ref. ^2^). The fundamental reason for causing the opposite result is that unlike 3D metal Pd, the 2D metal 1T′-MoTe_2_ can suppress the strong Fermi pinning effect at the metal-semiconductor interface. In reality, the Schottky barrier heights between most 3D metals and monolayer MoS_2_ had seriously deviated from the Schottky–Mott rule^22,24^, resulting in the significantly lower Schottky barrier of 0–0.3 eV than the theoretical values in Fig. 2e. Therefore, followed by the formula (1), the ideal value of the slope $S = \left| {d {{\varPhi}} _{\mathrm{SB}}/d{{\varPhi}} _M} \right|$ in Fig. 2e is 1. However, the *S* value of the Schottky barrier height between 3D metals and monolayer MoS_2_ is only 0.09 which is much lower than the ideal value, indicating the Fermi levels of the MoS_2_ in these metal-semiconductor interfaces are severely pinned.

The Fermi pinning effect is highly related to the defect-induced gap states of the semiconductors, the additional defects and chemical disorders involved by device fabrications, and the metal-induced gap states^2,24^. The benefits of using 2D metals in suppressing the Fermi pinning effect are as follows. Firstly, lattice structure damages of the channel materials from the 3D metal evaporation are avoided. Secondly, some chemical disorders in the interface will not be involved by various solvents used in lithography processes. Thirdly, unlike 3D metals, 2D metals can suppress the metal-induced gap states formed from the decaying metallic wavefunction of the 3D metal itself^24^.

To explore the dependence of the Schottky barrier height on the performance of 2D semiconductor-based diodes, we compared the rectifying performance of the Pd/MoS_2_ and 1T′-MoTe_2_/MoS_2_ diodes. According to the aforementioned discussions, the band diagrams of the two diodes with different Schottky barrier heights are shown in Fig. 2f. The 1T′-MoTe_2_/MoS_2_ diode shows good rectifying behavior, and its rectifying ratio reached ~101 at *V*_DS_ = ± 1 V (Fig. 2g). While the rectifying ratio of the Pd/MoS_2_ diode is only 11 at *V*_DS_ = ± 1 V, which is still consistent with the reported values of 4–30 of the Pd/MoS_2_ diodes (Supplementary Table 1). This performance comparison shows that a large Schottky barrier height is essential for building a high-performance Schottky diode.

### Widening of Schottky barrier width

To construct an excellent 2D semiconductor-based Schottky diode, in addition to a large Schottky barrier height, a spacious Schottky barrier width is also essential. When the Schottky barrier width is very tiny, the tunneling possibility will significantly increase^18^, resulting in the large reverse tunneling current of diodes. The Schottky barrier width *W* of the Schottky junction can be obtained by following formula^3^:

2 $$W = \sqrt {\frac{{2\varepsilon _sV_{bi}}}{{qN_A}}}$$

where $\varepsilon _s$ is the dielectric constant of semiconductors, $V_{{bi}} = ({{\varPhi}} _M - {{\varPhi}} _S)/q$ is the built-in potential, *q* is the elementary charge, and *N*_*A*_ = *N*_2D_/*d*_MoS2_ is the body charge concentration of the channel materials. The smaller Schottky barrier width of the 2D semiconductor-based Schottky diodes can be attributed to the high carrier concentration of >10^19^ cm^−3^ of the ultra-thin physical bodies. Generally, the n-type doping of monolayer MoS_2_ is considered to mainly originate from the electron doping induced by widespread sulfur vacancies (SVs)^33,34^.

**Fig. 3:**
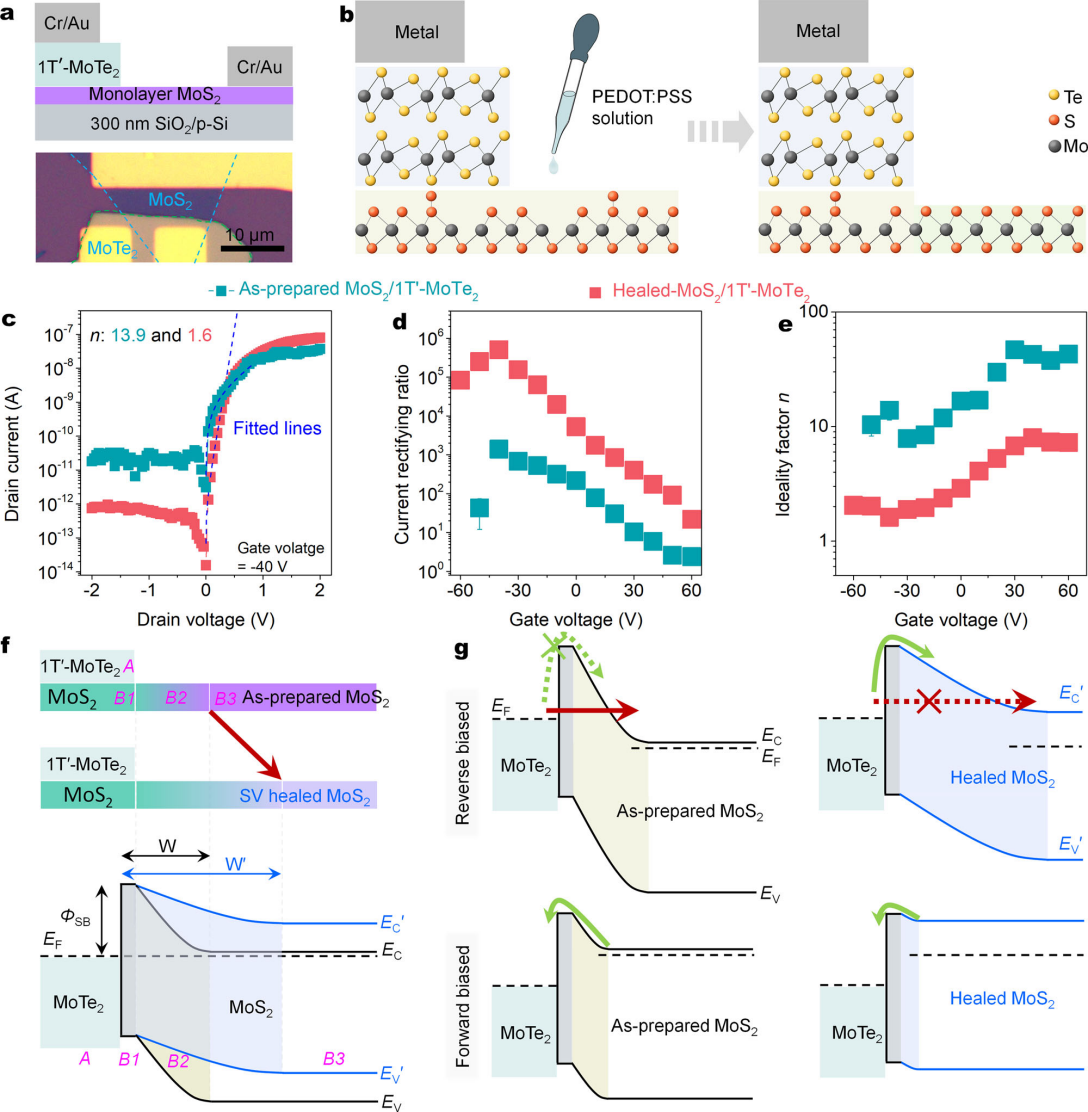
Giant optimization of the Schottky junction through thickening the Schottky barrier width. **a** Schematic diagram (top) and optical micrograph (bottom) of 1T′-MoTe_2_/MoS_2_ Schottky junctions in which the monolayer MoS_2_ serves as the transport channel, p-Si is the control gate, SiO_2_ layer is the gate dielectric, the 1T′-MoTe_2_ is the bias drain electrode, and the Cr/Au is grounded. **b** Schematic diagram of the atomic structure of the 1T′-MoTe_2_/MoS_2_ Schottky junctions before and after the SVSH. **c** Output curves in logarithmic scales of the 1T′-MoTe_2_/MoS_2_ Schottky junctions before and after the SVSH. **d**, **e** Gate-dependent current rectifying ratios (**d**) and ideality factors (**e**) extracted from Supplementary Fig. 8 before and after the SVSH. **f** Device schematic diagrams (top) and band structure diagrams (bottom) before and after the SVSH. The *E*_*F*_, *E*_*C*_, and *E*_*V*_ are Fermi level, conduction band, and valence band, respectively. The A, B1, B2, and B3 regions refer to the metal, contacted MoS_2_, depletion exposed MoS_2_, and pure channel MoS_2_. The yellow and blue gradient areas mean the depletion regions of the Schottky junctions. It should be noted that to simplify the band structure, the tunneling barriers originated from the vdWs gap between 1T′-MoTe_2_ and MoS_2_ are intentionally removed in the whole manuscript. **g** Band structure diagrams of the two-state devices at forward and reverse biased voltages. The green and red arrows (solid/dashed: main/almost-impossible style) indicate the thermionic emission current and thermally assisted tunneling current, respectively. The movement direction of an electron during reverse (forward) biased voltage is from the MoTe_2_ to the MoS_2_ (MoS_2_ to MoTe_2_).

To further improve the quality of the 2D semiconductor-based Schottky junctions and suppress its reverse leakage current (Fig. 3a), our previous acid-induced sulfur vacancy self-healing (SVSH) effect was employed to heal the SVs of the MoS_2_ in Fig. 3b (ref. ^28^). The treatment operation details and healing mechanism are also presented later (‘Methods’ and Supplementary Fig. 5a). Scanning transmission electron microscopy (STEM) shows the S/Mo ratio of monolayer MoS_2_ has been increased from ~1.85 to ~1.92 in Supplementary Fig. 5b–d, which are roughly consistent with the results (from ~1.84 to ~1.93) measured by X-ray photoelectron spectroscopy (XPS) in Supplementary Fig. 6. The removal of SVs makes the threshold voltage of the MoS_2_ FET close to zero in Supplementary Fig. 7d, illustrating the intrinsic electron concentration is significantly lowered by 3.9 times from 2.51 × 10^12^ to 6.46 × 10^11^ cm^−2^. The lowering of electron concentration is double confirmed by the increase of work function from ~4.3 to ~4.6 eV measured through the ultraviolet photoelectron spectroscopy (UPS) in Supplementary Fig. 7e.

The decrease of the electron concentration *N*_*A*_ has efficiently increased the Schottky barrier width of the 1T′-MoTe_2_/MoS_2_ Schottky junctions from ~2.9 to ~4.6 nm (calculations in ‘Methods’). As expected, the SVSH effect significantly shrinks the reverse leakage current from 2 × 10^−11^ to 8 × 10^−13^ A, while roughly maintaining the forward current (Fig. 3c). The 1T′-MoTe_2_/as-prepared MoS_2_ Schottky junction shows the obvious rectifying behavior and delivers the maximum rectifying ratio of ~1.4 × 10^3^ at *V*_D_ = ± 2 V and *V*_G_ = −40 V in Fig. 3d. This performance is consistent with the general value (10–10^4^) of the 2D semiconductor-based Schottky junctions reported in the literature (Supplementary Table 1). However, the current rectifying ratio of the 1T′-MoTe_2_/healed MoS_2_ Schottky junction is enhanced from ~364 times to ~5.1 × 10^5^ at *V*_D_ = ± 2 V and *V*_G_ = −40 V in Fig. 3d. This ratio is the best performance among monolayer 2D semiconductor-based Schottky diodes reported to date (Supplementary Table 1). The gate-dependent output curves and transfer curves of the Schottky junction before and after the SVSH are presented in Supplementary Fig. 8. To minimize the performance degradations from transferring monolayer MoS_2_ in the device fabrication, another device design has been abandoned, in which the MoS_2_ is top on the metallic 1T′-MoTe_2_. Though the abandoned device design can achieve the SV healing of the MoS_2_ above the 1T′-MoTe_2_, the performance degradations from transferring MoS_2_ may offset the benefits of defect healing.

To further evaluate the rectifying performance of the 1T′-MoTe_2_/MoS_2_ Schottky junction diode, an ideality factor (*n*) was estimated at a small forward bias (here is 0.02–0.35 V, Fig. 3c) by fitting to the Schottky diode equation^13,28,35^:

3 $$I_{\mathrm{D}} = I_{\mathrm{S}}\left[ {{\mathrm{exp}}\left( {\frac{{V_{\mathrm{D}}}}{{nV_{\mathrm{T}}}}} \right) - 1} \right]$$

where *I*_D_, *I*_S_, *V*_D_, and *V*_T_ denote drain current, reverse leakage current, drain voltage, and thermal voltage, respectively. As the gate voltage sweep from positive to negative in Fig. 3e, the ideality factor derived from the parameters of the fitting equation gradually approaches the perfect limit value of 1 and gain ~1.6 at *V*_G_ = −40 V (the fitted lines of other gate voltages in Supplementary Fig. 8a, b), indicating that the Schottky junction probably reaches a near-ideal diode behavior under the dual effect of the atom-vacancy healing and gate doping^2,13,25^. This similar performance improvement effect can be reproduced in dozens of devices. Besides, with gate voltage sweeping from positive to negative, both the rectifying ratios and ideal factors will gain the best performance at some negative gate voltage and then start to degrade. This performance transition may be related to the trade-off between the resistances of the bare Schottky junction (consist of region A, B1, and B2 in Fig. 3f) and the series resistances of the MoS_2_ channel (region B3)^36^. The Schottky junction dominates the total resistance of the diode above the transition voltage. While the interference series resistance of the MoS_2_ channel will gradually increase its proportion in total resistance and replace the Schottky junction to dominate the diode after crossing the transition voltage.

To remove the interferences from electrode contacts of the MoS_2_/Cr and 1T′-MoTe_2_/Cr interfaces, both MoS_2_ and MoTe_2_ transistors before and after the PEDOT:PSS treatment was also fully characterized. The nearly linear current–voltage relationships in the output curves of the Cr-MoS_2_-Cr transistors before and after the SVSH are observed in Supplementary Fig. 7b, c, indicating that the Ohmic contacts of the MoS_2_/Cr interfaces have not been adversely affected. It should be noted that the Cr/MoS_2_ interfaces are not truly Ohmic contacts^20^, and its Schottky barrier heights before and after the SVSH are largely maintained at ~0.1 eV (Supplementary Fig. 9). The Schottky barriers of the Cr/MoS_2_ interfaces are believed to be highly related to the formation of CrS_x_ (ref. ^37^). On the other hand, compared to the untreated one, the treated 1T′-MoTe_2_ transistor still maintains a linear current–voltage relationship and constant conductance in Supplementary Fig. 7f–h, indicating that the variations on the 1T′-MoTe_2_ side from the acid treatment are almost negligible. Therefore, the main action object of the PEDOT:PSS treatment is the metal–semiconductor interface of the 1T′-MoTe_2_/MoS_2_ Schottky junction, rather than the other electrode contacts.

While reducing the electron doping of the SVs of the covered MoS_2_ (region B2) in Fig. 3f only will efficiently enlarge the depletion width of the Schottky barrier but will not significantly alter the Schottky barrier height ${{\varPhi}} _{\mathrm{SB}}$. The variable temperature transport studies show the Schottky barrier heights ${{\varPhi}} _{\mathrm{SB}}$ before and after treatment are calculated as 501 ± 26 and 510 ± 19 meV in Supplementary Fig. 4, suggesting the SVSH effect can not largely alter the Schottky barrier height ${{\varPhi}} _{\mathrm{SB}}$. On the other hand, if the Schottky barrier height ${{\varPhi}} _{\mathrm{SB}}$ is considered to be significantly enhanced by the SVSH effect, both the forward and reverse biased currents will be significantly reduced^38^. However, the forward-biased currents are nearly unchanged or even increased in the partial negative voltage region in Supplementary Fig. 8c right. Besides, previous studies showed that not doping the covered region B1 but doping the exposed region B2 (Fig. 3f) could also significantly modulate the Schottky barrier width^26^. In reality, even if the monolayer MoS_2_ of the covered region B1 (Fig. 3f) was healed, the improvement effect induced by the SVSH on the rectifying behavior of the 1T′-MoTe_2_/MoS_2_ Schottky diode would not be significantly changed (Supplementary Fig. 10).

### Improvement mechanism of the rectifying performance

So how does enlarging the Schottky barrier width improve the rectifying behavior? Based on this band structure transformation of the Schottky junction in Fig. 3f, the depletion region width of the as-prepared Schottky junction under reverse bias is too narrow to easily generate large tunneling current Fig. 3g. When the Schottky barrier height remains roughly constant in Fig. 3g, enlarging the Schottky barrier width can transform the charge injection style under reverse bias from thermionic emission to thermionic field emission (also call thermally assisted tunneling)^17^. Thus, different from the 1T′-MoTe_2_/as-prepared MoS_2_ Schottky junction, the reverse leakage currents of the optimized Schottky junctions are significantly suppressed. But in the forward bias, an electron only needs to pass through the Schottky barrier with a small built-in potential to reach the metal electrode 1T′-MoTe_2_ in Fig. 3g bottom. On the other hand, as forward bias is added, the resistance of the bare Schottky junction decreases, the negative effect of the series resistance of the MoS_2_ channel (region B3) becomes comparable. To put it simply, the total resistance of the 1T′-MoTe_2_/healed MoS_2_ Schottky junction in the forward bias can be presumably assumed to be divided into the large MoS_2_ series resistance (region B3) and small bare Schottky junction resistance (region A, B1, and B2). While the total resistance of the 1T′-MoTe_2_/as-prepared MoS_2_ Schottky junction is the sum of the small MoS_2_ series resistance and large bare Schottky junction resistance in Fig. 3g bottom-left. In this case, the total resistance of the two Schottky junctions in the forward bias may be close in the most 1T′-MoTe_2_/MoS_2_ Schottky junctions, which has been confirmed by the two similar forward currents in Fig. 3c and Supplementary Fig. 8d. Based on the performance survey of the dozens of Schottky junctions, the differences between the two forward currents of the as-prepared and healed devices are fluctuated by the MoS_2_ channel length. In a word, the decline of *E*_*F*_ induced by the SVSH can greatly reduce the reverse leakage current, roughly maintain the forward current, and finally improve the rectifying ratio and ideal factor.

### Optimization of other Schottky junctions

**Fig. 4:**
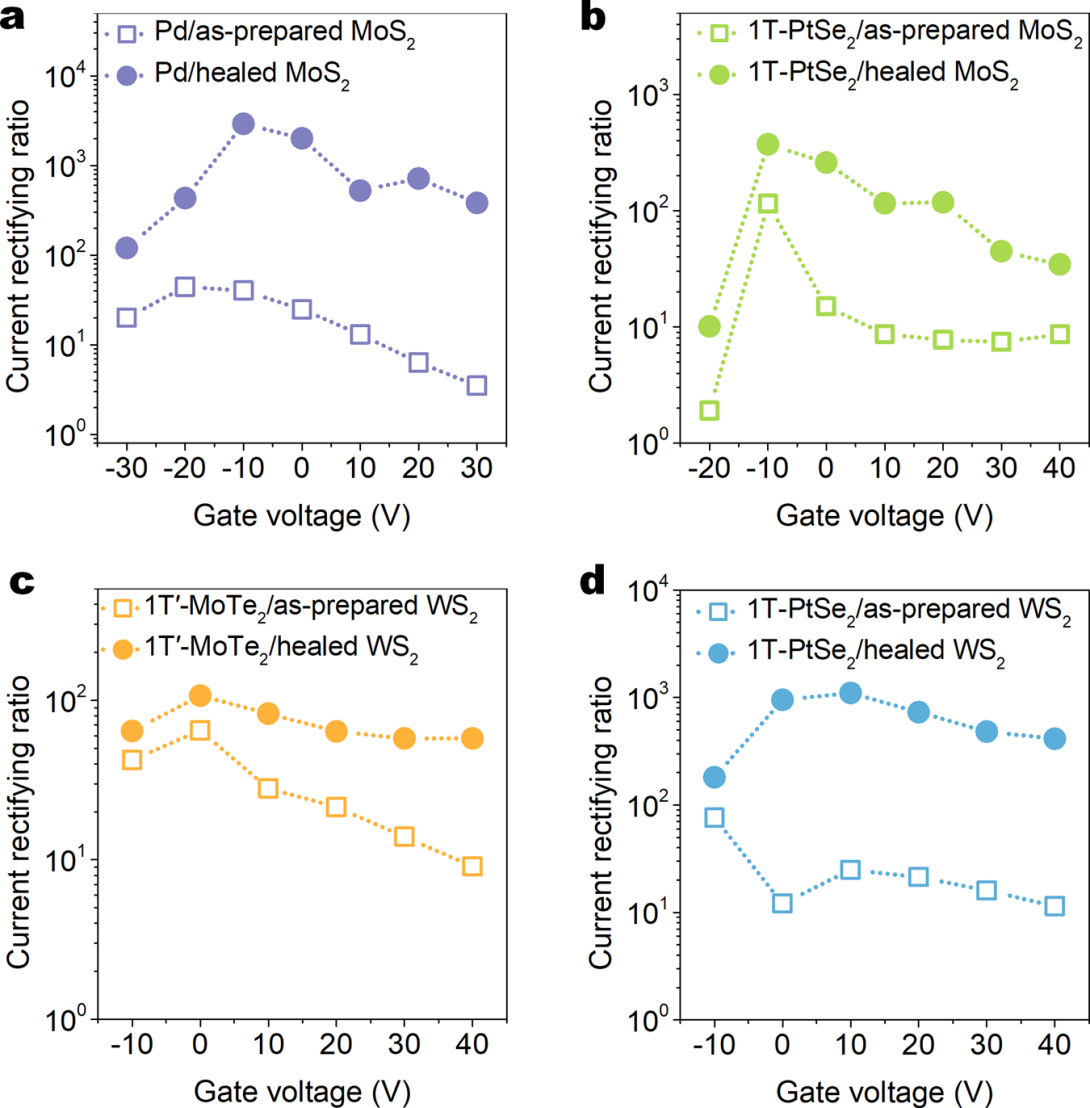
Generalization of performance optimization strategy of thickening Schottky barrier width. **a**–**d** Gate-dependent rectifying ratios of the Pd/MoS_2_ (**a**), 1T-PtSe_2_/MoS_2_ (**b**), 1T′-MoTe_2_/WS_2_ (**c**), and 1T-PtSe_2_/WS_2_ (**d**) metal-semiconductor diodes extracted from Supplementary Fig. 11 before and after the SVSH. It should be noted that both 1T′-MoTe_2_ and 1T-PtSe_2_ are multilayer over 10 nm and behave metallic characteristics to the only function as electrodes.

Except for the 1T′-MoTe_2_/MoS_2_ Schottky diode, the performance optimization strategy induced by the vacancy healing can also be generalized to other MoS_2_-based diodes. The monolayer MoS_2_ Schottky diodes with the Pd and Cr asymmetric metal electrodes after the solution treatment can only exhibit a maximum rectifying ratio of ~2.9 × 10^3^ in Fig. 4a, which are considerably worse than that of the 1T′-MoTe_2_/healed MoS_2_ Schottky junctions in Fig. 3d. The poor performance of the Pd/MoS_2_ Schottky junction can be partially attributed to its small Schottky barrier height of ~0.12 eV (ref. ^2^), which is lower than the value of ~0.5 eV of the 1T′-MoTe_2_/healed MoS_2_ Schottky junction in Fig. 2d. Nevertheless, this optimized performance of the Pd/MoS_2_ Schottky junction is much higher than that (1~100) of similar MoS_2_-based Schottky junctions with evaporated asymmetric metal electrodes (Supplementary Table 1). As mentioned before, the Pd/MoS_2_ Schottky junctions may be suffered from the severe Fermi pinning effect in the interface between the evaporated Pd electrode and the monolayer MoS_2_. Thus, the origin of the superior rectifying behaviors of the 1T′-MoTe_2_/healed-MoS_2_ Schottky junctions can be attributed to the dual effect with the large Schottky barrier height and the spacious Schottky barrier width.

Similar to the 1T′-MoTe_2_/MoS_2_ and Pd/MoS_2_ Schottky junction, the rectifying ratios of the 1T-PtSe_2_/MoS_2_ also were systemically enhanced by the SVSH in Fig. 4b. Due to the similar layered structure between transition metal dichalcogenides (TMDCs) materials, the acid-induced vacancy healing effect has been confirmed to apply to other TMDCs semiconductors^39^. Thus, we also generalize the performance optimization strategy to other TMDCs-based Schottky junctions (Supplementary Fig. 11 and Fig. 4c, d). Similarly, the vacancy healing effect can also increase the rectifying ratios of the 1T′-MoTe_2_/WS_2_ and 1T-PtSe_2_/WS_2_ Schottky junctions, which means that their Schottky barrier widths may be greatly enhanced. It should be noted that similar to the 1T′-MoTe_2_ film, the 1T-PtSe_2_ transistor still maintains a linear current–voltage relationship and constant metallic conductance in Supplementary Fig. 12.

### Photoresponse characterization of the Schottky junctions

**Fig. 5:**
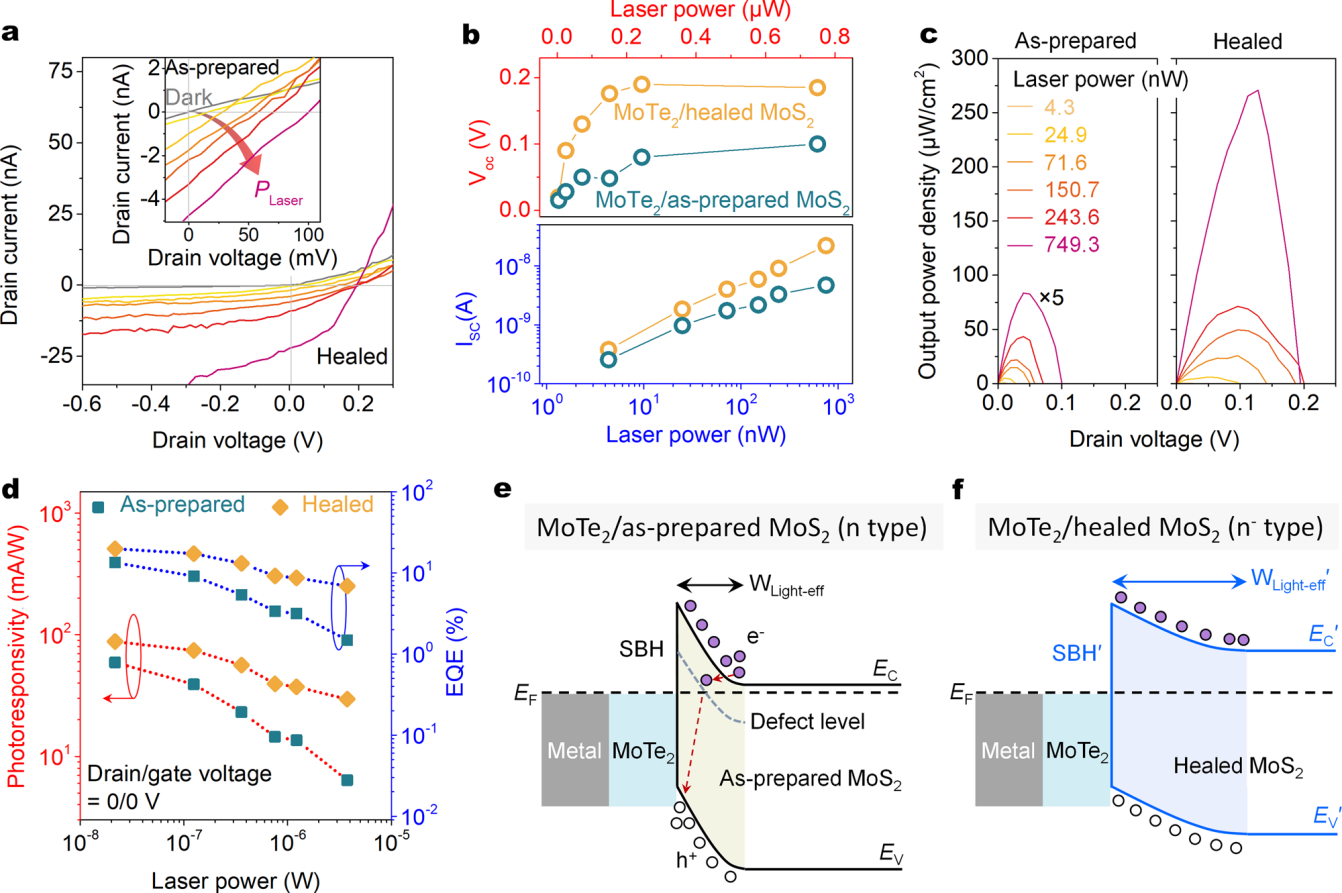
Photoresponse optimization of the 1T′-MoTe_2_/MoS_2_ Schottky junction photodiodes. **a** Output curves of the 1T′-MoTe_2_/healed MoS_2_ and 1T′-MoTe_2_/as-prepared MoS_2_ (inset) Schottky junctions in dark and under 532 nm light irradiation with various laser power densities (*P*_Laser_). The gate voltages before and after the SVSH are zero. **b** Short circuit current (*I*_SC_) and open-circuit voltage (*V*_OC_) as a function of *P*_Laser_ extracted from (**a**). **c** Output electrical power of the as-prepared (left) and healed (right) devices as a function of drain-source voltage. **d** *P*_Laser_-dependent photoresponsivities (red) and EQE (blue) at zero bias. **e**, **f** Band structure diagrams of the 1T′-MoTe_2_/MoS_2_ Schottky junctions before (**e**) and after (**f**) the SVSH under light irradiation. The purple and hollow circles represent electrons and holes, respectively. The red dashed arrow refers to the electron movement direction. The yellow and blue gradient areas refer to the depletion regions of the Schottky junctions. *W*_Light-eff_ refers to the efficient light absorption width of the Schottky junction.

Next, we focus on the photoresponse of the 1T′-MoTe_2_/MoS_2_ Schottky junctions before and after the SVSH and evaluate its performance in photovoltaic detectors in the visible wavelength range. Figure 5a shows that regardless of the as-prepared device or the healed device, the short-circuit current *I*_SC_ and open-circuit voltage *V*_OC_ show a monotonic increase with the increase of laser power density. Under the same laser power density, both the *I*_SC_ and *V*_OC_ are significantly increased by the SVSH effect. As a comparison, the obvious photovoltaic response is not observed in the as-prepared and healed MoS_2_ transistors (Supplementary Fig. 13), illustrating that the electrode contacts of the Cr/MoS_2_ can not donate the photoresponse at zero biased voltage. Besides, the efficient interface charge separation of the 1T′-MoTe_2_/MoS_2_ Schottky junction has been confirmed by the PL spectra (Supplementary Fig. 14). With the power intensity increasing, the *I*_SC_ near linearly increases, and the *V*_OC_ will gradually enter the saturated state (Fig. 5b). In the 1T′-MoTe_2_/healed-MoS_2_ Schottky junction, a high *I*_SC_ of ~22.2 nA and a large *V*_OC_ of ~0.19 V are obtained under the power density of 8.23 mW/mm^2^ (junction area of ~671 μm^2^). Under the maximum laser power density, the output electrical power density is increased by about 16 times from ~16.7 to ~270.7 μW/cm^2^ through the SVSH strategy in Fig. 5c.

To evaluate the performance improvement of the MoTe_2_/MoS_2_ photodetector, the photoresponsivity and external quantum efficiency (EQE) are systemically characterized. The photoresponsivity is defined as *R* = *I*_ph_/(*P*_in_·*A*), where *I*_ph_ is the photocurrent, *P*_in_ is the incident laser power density, and *A* is the junction area of the 1T′-MoTe_2_/MoS_2_ diode. EQE is the ratio of collected charge carriers to incident photons and can be calculated by EQE = (*I*_sc_·*h*·c)/(*e*·*λ*·*P*_in_·*A*), where *h*, c, and *λ* are the Planck’s constant, the speed of light, and the wavelength of the incident laser, respectively^40^. At laser power density of ~1.9 mW/mm^2^ (the laser spot size is ~2.25 μm^2^), the photoresponsivities of the as-prepared and healed devices are ~59/88 mA/W and EQE of ~13/~20% in Fig. 5d.

This improvement in photoresponse performance can be attributed to the SVSH effect which greatly enlarges the Schottky barrier width, meaning that the photoresponse area is increased in Fig. 5e, f. The second possible reason is that the sub-bandgap defect states of SVs will trap the photo-generated electrons and promote non-radiative recombination in Fig. 5e, leading to lower *I*_SC_ and *V*_OC_ (ref. ^41^). Thirdly, a neglected but reliable reason is that the removal of electron doping induced by the SVSH effect greatly improves the light absorption efficiency of monolayer MoS_2_ to form more photocarriers^42,43^. The electron doping removal induced by gate doping also can enhance the *I*_SC_ and *V*_OC_ of the MoS_2_ Schottky junction^2^, while our improvement strategy of defect healing is gate-free and doesn’t rely on the external gate voltage.

## Discussion

In conclusion, a near-ideal 1T′-MoTe_2_/MoS_2_ Schottky diode with a large Schottky barrier height was successfully fabricated by stacking the 2D metallic 1T′-MoTe_2_ and the semiconducting monolayer MoS_2_. We systemically demonstrate that besides the large Schottky barrier height, thickening the tiny Schottky barrier width can further improve their performance of 2D Schottky junctions by healing the original defects in the 2D semiconductor counterparts. We further confirm that this Schottky barrier optimization strategy is generally applicable to other Schottky diodes, including 1T-PtSe_2_/MoS_2_, 1T′-MoTe_2_/WS_2_, 1T-PtSe_2_/WS_2_, and Pd/MoS_2_. This work provides a proof-of-concept performance optimization strategy for building high-performance Schottky diodes.

## Methods

### Growth of monolayer MoS_2_

The monolayer MoS_2_ films were grown by the atmospheric pressure oxygen-assisted CVD method^28,35^. MoO_3_ (Sigma-Aldrich, ≥ 99.5% purity) and sulfur (Sigma-Aldrich, ≥ 99.5% purity) were applied as the precursor and reactant materials, respectively. MoO_3_ powder (10 mg) was placed in a quartz boat at the center of a single zone furnace (Tianjin Zhonghuan Electrical Furnace Co., Ltd.). A 2 × 2 cm^2^ Si substrates with 300 nm SiO_2_ (from Silicon Valley Microelectronics, Inc.) were put face down at top of the MoO_3_ powder. S powder was heated to 170 °C by the home-made heating belt and carried through a high-purity Ar flow of 500 sccm. The experiments were implemented at a reaction temperature of 850 °C for 30 min. When the temperature reached the set value of 850 °C, the oxygen of ~1.2 sccm was introduced as the auxiliary growth gas and it was kept for 30 min. Finally, the monolayer MoS_2_ samples were cooled naturally under S vapor to minimize SVs.

### Thermal CVD growth of WS_2_

The WS_2_ films were grown by the typical thermal CVD process^44^. To prepare the WS_2_ flake single crystals, an alumina boat loaded with WS_2_ (Alfa Aesar, 99.8% purity) powder (3–5 *g*), was placed into the middle heating zone of the single zone furnace (from Therm craft XST-2-0-12-MINI-240). The Si substrates (with 300 nm SiO_2_) were placed on the quartz boat around the outlet of the furnace. The deposition was performed at the temperature of ~1180 °C and 150 sccm high-purity Ar flow for 1–3 min.

### Material preparation and device construction

The metal phase 1T′-MoTe_2_ and 1T-PtSe_2_ films were obtained by mechanical exfoliation of the 1T′-MoTe_2_ (from Hefei Kejing Co.) and 1T-PtSe_2_ (from 2D Semiconductors) bulks. The devices were constructed by stacking CVD-grown monolayer MoS_2_ and exfoliated 1T′-MoTe_2_ flakes with the accurate transfer platform (Metatest, E1-T). We used a standard electron beam lithography (EBL) to pattern source/drain contacts of MoS_2_ and 1T′-MoTe_2_ FETs, followed by thermal beam evaporation of 10/50-nm-thick Cr/Au electrodes and lift-off. The metallization was performed by a thermal evaporation system (KYKY Technology), and the thermal evaporation pressure was 1–5 × 10^−5^ Pa. Especially, our electrode contact optimization for CVD-grown monolayer MoS_2_ FETs was the mild Argon plasma etching which can remove residual polymethyl methacrylate (PMMA, A8, Mircochem Inc.) and produce a small amount of SVs. The plasma etching parameters (from Tailong Electronics RIE 100) are the radio-frequency (13.56 MHz) power of 5 W, the Ar flow of 10 sccm, and the treatment duration of 35–45 s. Both the ~15/45 nm Pd/Au electrodes and the ~15/45 nm Cr/Au electrodes in the Schottky junction diodes of the asymmetric metal electrodes were deposited by thermal evaporation.

### Acid solution treatment for sulfur vacancy self-healing (SVSH)

Firstly, the MoS_2_ sample was immersed in the undiluted poly(3,4-ethylenedioxythiophene):poly(4-styrenesulfonate) (PEDOT:PSS) solution (Sigma-Aldrich, 1.0 wt%) at room temperature, after standing for 5 min, and then immersed in plenty of deionized (DI) water to wash the water-soluble PEDOT:PSS solution for 30 min. The quality of the DI water, which was used to rinse the samples (~0.8 × 0.8 cm^2^ Si substrates), is >200 + >200 mL to the twice cleaning. Further, the residual deionized water was dried with nitrogen flow. Both Raman spectra and gas chromatography–mass spectrometry (GC–MS) can prove that there is no residual PEDOT:PSS in the 1T′-MoTe_2_/healed MoS_2_ Schottky diodes (Supplementary Figs. 7f and 15).

### PL and Raman measurements

PL and Raman spectrum measurements were performed with confocal microscopy (JY-HR800 or Renishaw inVia Qontor) under 532 nm laser at room temperature and in the ambient atmosphere. It should be noted that to reduce the measurement error of the 1T′-MoTe_2_ film from strong laser irradiation, both the PMMA package and the weak laser power density (<5 mW/μm^2^) are simultaneously arranged^45^.

### AFM and KPFM measurement

The AFM and KPFM images were obtained via the Bruker Icon AFM system. An AFM tip (SCM-PIT-V2, Bruker Nano Inc., USA) was selected to probe the topography and thickness of the samples under the peak-force working mode, while the AM-KPFM measurements were measured under the tapping mode via the same tip of SCM-PIT-V2 with a resonant frequency of 75 kHz, a tip radius of 25 nm, a Pt–Ir coated conductive tip, a tip-sample height of 100 nm, and the ac bias of 0.5 V on a grounded substrate. These tests were performed in an atmospheric environment.

### Calculation of Schottky barrier width *W*

The Schottky barrier width *W* of the Schottky junction can be obtained by Eq. (2)^3^. We use a relative dielectric constant of 3.3 for MoS_2_ (ref. ^46^). The ${{\varPhi}} _M$ of 1T′-MoTe_2_ and the ${{\varPhi}} _S$ of 1H-MoS_2_ before and after the SVSH effect can be extracted from Supplementary Fig. 3c, d and Supplementary Fig. 6e. The *d*_MoS2_ is the thickness of 0.65 nm of monolayer MoS_2_. Thus, the Schottky barrier widths *W* before and after the SVSH effect are ~2.9 and ~4.6 nm, respectively.

### STEM measurement

STEM samples were prepared using a standard PMMA-based transfer, where the samples were spin coated and soaked in a KOH solution (5 wt%). Subsequently, acetone (AR, from RCI Labscan Limited) and isopropyl alcohol (AR, from Aladdin) were used to clean the TEM grid, allowing for the suspension of the films in the solution and the transfer to TEM grids. All STEM samples were baked at 160 °C for 5 h under vacuum (the pressure was <1 Pa) before the microscopy experiment and characterized on a JEM-ARM200F TEM operating at 80 kV. The STEM images were prepared by partially filtering out the direct spot in the FFT of the image, which helps to remove the contrast generated by the surface contaminations (Supplementary Fig. 5b, c top). After these filtering processes, the STEM images are converted into the plain text signal, in which the signal of the Mo atoms was accurately eliminated by a home-made program. Finally, the program-processed data was produced into the filtered STEM images (Supplementary Fig. 5b, c bottom) by using an Origin Pro 9.0. It should be noted that to reduce the interference of ex-situ measurement, the CVD-grown monolayer MoS_2_ samples in the STEM measurement were originated from the same SiO_2_/Si substrate. For example, a 2 × 2 cm^2^ Si substrates with CVD-grown monolayer MoS_2_ were evenly cut into two groups. Generally, the defect concentrations of the two sets of samples can be roughly considered the same.

### XPS measurement

XPS was carried out using an ESCALab250 electron spectrometer from Thermo Scientific Corporation with monochromatic 150 W Al Kα radiation (hν = 1486.7 eV). The take-off angle for XPS was 90°, and the pass energy for the narrow scan was 30 eV. The base pressure was about 6.5 × 10^−10^ mbar. The analyzer of the spectrometer was calibrated using sputter cleaned Au, Cu, and Ag foils, as is outlined in the GB/T 19500-2004 standard^47^. In this calibration method, the standard calibrated binding energies of the Au, Cu, and Ag standard samples are Au 4*f*_7/2_ of 84.0 ± 0.1 eV, Cu 2*p*_3/2_ of 932.7 ± 0.1 eV, and Ag 3*d*_5/2_ of 368.3 ± 0.1 eV, respectively. To compensate for sample charging, the XPS spectra were calibrated using the Si 2*p*^3/2^ peak of the SiO_2_ substrate at 103.6 eV as a reference. For XPS peak analysis and deconvolution, the software Avantage 4.15 was employed, where a mixed Gaussian–Lorentzian function and an active Shirley background were used for peak fitting. The S/Mo ratios were determined from the integrated areas of the S 2*p* and Mo 3*d* peaks factored by their corresponding relative sensitivity factors of 0.668 and 3.321. The error in the S/Mo ratios was obtained from the peak fitting residuals.

### UPS measurement

UPS curves were obtained in an ultrahigh vacuum chamber using a helium lamp source emitting (AXIS ULTRA DLD). The ultraviolet light source used was He I (21.22 eV) that has not been monochromated. The basic vacuum of the analysis chamber during the UPS analysis and testing of this equipment was 3.0 × 10^−8^ Torr. The biased voltage during the test was −9 V.

### GC–MS measurement

GC–MS was conducted on an Agilent 7890A/5975C installed with an HP-5MS column (30 m × 0.25 mm × 0.25 μm). The temperatures of both inlet and detection were set at 300 °C. The initial temperature of the oven was set at 30 °C and kept for 1 min, and then was programmed from 30 to 250 °C with a heating rate of 10 °C/min, and finally was retained at 250 °C for additional 5 min. This last increase was to clean the column from any residues. The MS was set as the positive ion model with the mass-to-charge searching region from 20 to 1024. The preparation process of the sample as follows: Firstly, after the 1T′-MoTe_2_/MoS_2_ Schottky diodes were constructed on the SiO_2_/Si substrate, the Si substrate was immersed in the absolute ethanol (AR, from Honeywell International Inc.) solution of 3 mL for 2 h. Secondly, remove and blow-dry with nitrogen. After the sample was soaked in the PEDOT:PSS solution for 5 min, was rinsed it twice with plenty of deionized water (>200 + >200 mL) and was dried with nitrogen flow. Thirdly, repeat step #1. The two different absolute ethanol solutions before and after the PEDOT:PSS treatment were extracted 1 μL for the GC–MS test.

### Electrical measurement

The electrical characteristics were implemented by a semiconductor analysis system (Keithley 4200 and B2902 A). Electrical measurements were performed in a vacuum probe station (Lakeshore TTP4) with liquid nitrogen and base pressure ~10^−5^ Torr.

## Supplementary information

Supplementary information

## Data Availability

All data supporting this study and its findings are available within the article and its Supplementary Information or from the corresponding author upon reasonable request.

## Code availability

The codes that support the findings of this study are available from the corresponding author upon reasonable request.
