# Supplementary material for: Near-ideal van der Waals rectifiers based on all-two-dimensional Schottky junctions
Source: Nat Commun. 2021 Mar 9;12:1522. doi: 10.1038/s41467-021-21861-6 (PMC7943806; doi:10.1038/s41467-021-21861-6)
Supplement: Supplementary file 1 — Supplementary information [file 41467_2021_21861_MOESM1_ESM.pdf]

Supplementary information for

Near-ideal van der Waals rectifiers based on all-two-dimensional Schottky junctions

Xiankun Zhang, Baishan Liu, Li Gao, Xiaozhi Liu, Huihui Yu, Junli Du, Jiankun Xiao, Yihe Liu, Lin Gu, Qingliang Liao, Zhuo Kang, Zheng Zhang*, Yue Zhang*

*Correspondence to: zhengzhang@ustb.edu.cn (Z.Z.), yuezhang@ustb.edu.cn (Y.Z.)

This PDF file includes:

Supplementary Figure 1. Angle-dependent polarized Raman intensities of few-layer 1T′-$MoTe_2$

Supplementary Figure 2. Morphology characterization of the 1T′-$MoTe_2$/MoS_2 Schottky junction.

Supplementary Figure 3. Work function characterization of the 1T′-$MoTe_2$ nanosheet.

Supplementary Figure 4. Schottky barrier heights of the 1T′-$MoTe_2$/MoS_2 Schottky junctions before and after the treatment.

Supplementary Figure 5. STEM images of monolayer MoS_2 before and after the treatment.

Supplementary Figure 6. XPS spectra of monolayer MoS_2 before and after the treatment.

Supplementary Figure 7. Electrical properties of the 1H-MoS_2 and 1T′-$MoTe_2$ transistors before and after the treatment.

Supplementary Figure 8. Basic electrical characterizations of the 1T′-$MoTe_2$/MoS_2 Schottky junctions.

Supplementary Figure 9. Schottky barrier heights of the Cr/MoS_2 contacts before and after the treatment.

Supplementary Figure 10. Rectifying performance comparisons of the two Schottky junctions with different metal-semiconductor contacts.

Supplementary Figure 11. Performance comparisons of several Schottky junctions before and after the SVSH.

Supplementary Figure 12. Electrical properties of the 1T-$PtSe_2$ electrodes before and after the treatment.

Supplementary Figure 13. Photoresponse characterization of the MoS_2 transistors.

Supplementary Figure 14. PL spectra of the 1T′-$MoTe_2$/MoS_2 Schottky junction interface.

Supplementary Figure 15. Removal of the residual PEDOT:PSS in the Schottky diodes.

Supplementary Table 1. Performance survey of 2D semiconductor-based Schottky diodes.

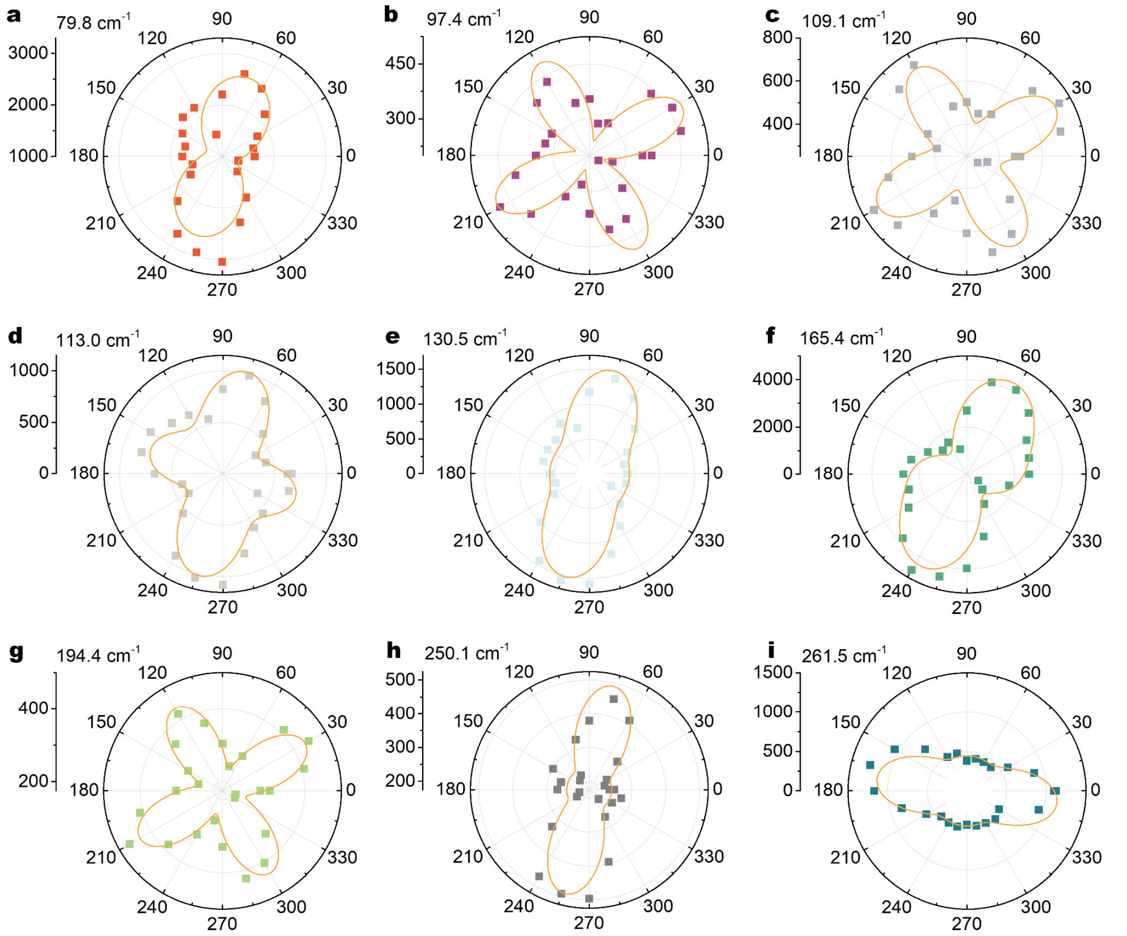

Supplementary Figure 1. Angle-dependent polarized Raman intensities of few-layer 1T′-$MoTe_2$. a-i, Intensities of 79.8 cm^{-1} (**a**), 97.4 cm^{-1} (**b**), 109.1 cm^{-1} (**c**),113.0 cm^{-1} (**d**), 130.5 cm^{-1} (**e**), 165.4 cm^{-1} (**f**), 194.4 cm^{-1} (**g**), 250.1 cm^{-1} (**h**), and 261.5 cm^{-1} (**i**) Raman modes as a function of angle, using a parallel-polarized laser. Dots and yellow solid curves are the experimental data and the best fits to the data for each Raman peak.

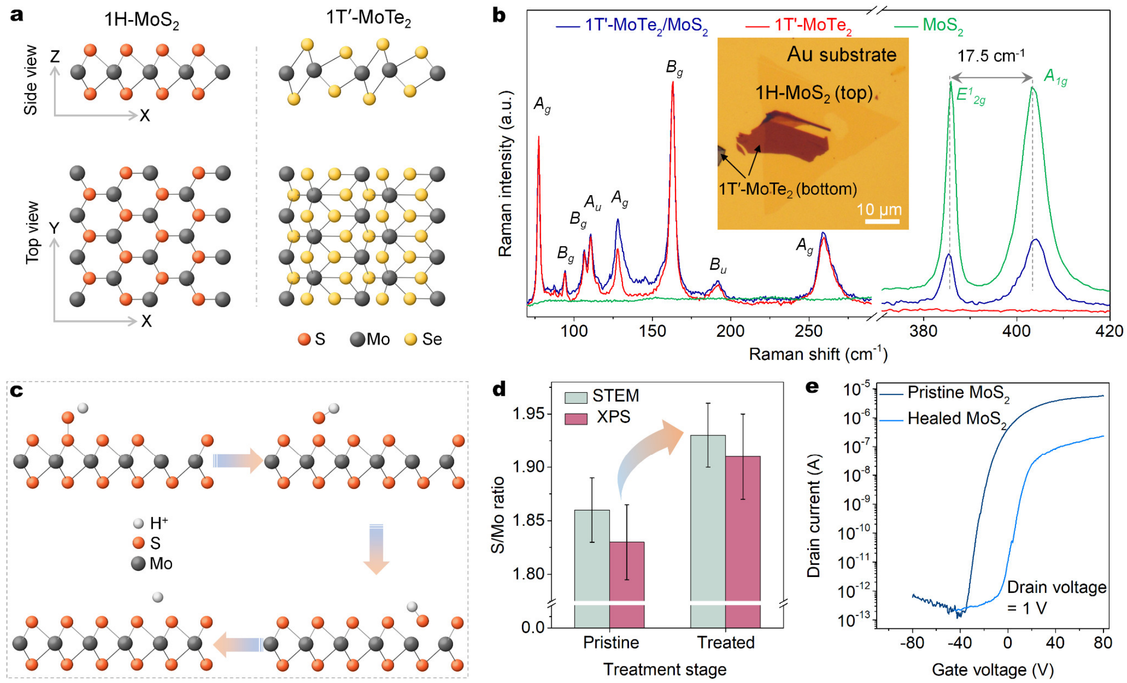

Supplementary Figure 2. Morphology characterization of the 1T′-$MoTe_2$/MoS_2 Schottky junction. **a**, Optical micrograph of the 1T′-$MoTe_2$/MoS_2 Schottky junction on the Au substrate. **b**, Raman spectra of the 1T′-$MoTe_2$/MoS_2 metal-semiconductor junction, 1T′-$MoTe_2$, and monolayer MoS_2. **c**, Corresponding height mapping. **d-e**, Height profile lines of the 1T′-$MoTe_2$ (**d**) and 1H-MoS_2 (**e**) films acquired from corresponding lines highlighted in **c**.

The exfoliated few-layer 1T′-$MoTe_2$ (bottom) and CVD-grown monolayer 1H-MoS_2 (top) are artificially stacked on a conductive substrate (60 nm Au deposited on p-Si) for basic characterization in Supplementary Fig. 2a. The thicknesses of $MoTe_2$ and MoS_2 are measured as ~10 and ~0.85 nm by atomic force microscope (AFM) in Supplementary Fig. 2c-e, indicating that the MoS_2 film is a single layer.

Raman spectroscopy was employed to characterize the components and interface coupling quality of the 1T′-$MoTe_2$/MoS_2 Schottky junctions in Supplementary Fig. 2b. Firstly, the frequency difference between A_{1g} and E^1_{2g} modes in Supplementary Fig. 2b is ~17.5 cm^{-1}, which also proves the MoS_2 film is a single layer[1, 2]. Secondly, compared to the isolated MoS_2, the E^1_{2g} and A_{1g} peaks of the MoS_2 in the overlapped region have been softened and stiffen to varying degrees. This feature indicates that there is a strong interlayer coupling effect at the 1T′-$MoTe_2$/1H-MoS_2 interface. Otherwise, these peaks won't shift[3]. The out-of-plane E^1_{2g} peak in the overlapped region exhibited a prominent redshift (~0.7 cm^{-1}) relative to that of the isolated MoS_2, which can be ascribed to the thermal lattice mismatch of the strong interlayer

coupling effect (or vdWs force) between the top and bottom layers[4]. The blue-shift of ~0.5 cm^{-1} of the in-plane vibrational mode A_{1g} is attributed to the occupation of anti-bonding states in the conduction band of MoS_2 by the electron concentration transfer from MoS_2 to 1T′-$MoTe_2$[5]. The occupation of anti-bonding states reduces the total electronic energy of the system, enhancing the Mo-S bonds and eventually stiffening the Raman mode.

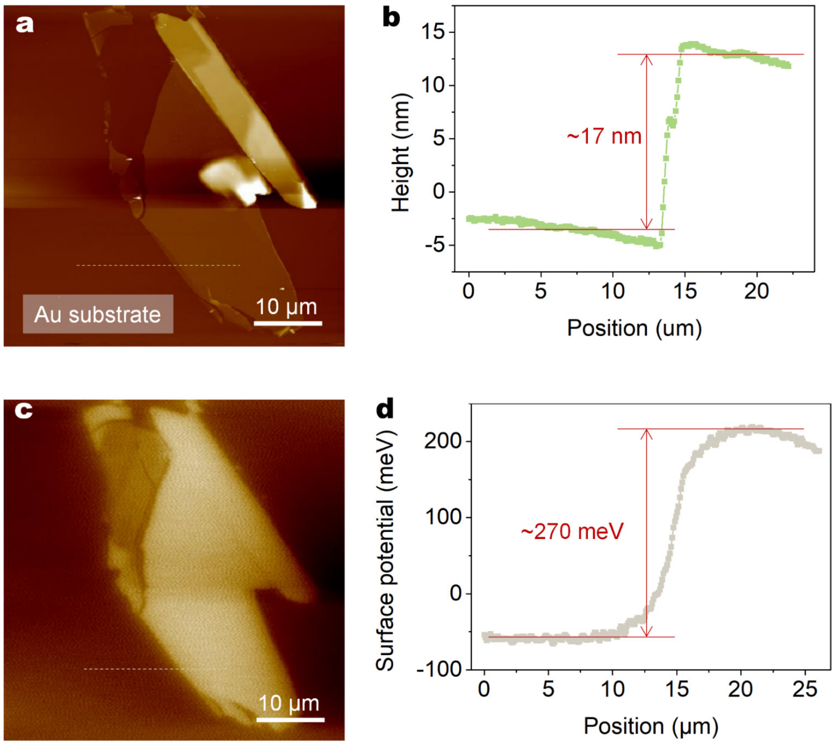

Supplementary Figure 3. Work function characterization of the 1T′-$MoTe_2$ nanosheet. a, c, The height mapping (**a**) and 2D surface potential image (**c**) of an exfoliated 1T′-$MoTe_2$ nanosheet. **b, d**, Height profile line (**b**) and surface potential cross-section (**d**) corresponding to green/gray dashed line in **a** and **c**. The difference in surface potential implies that the work function of 1T′-$MoTe_2$ is larger by ~270 meV than the Au substrate.

To further characterize the electronic structure of the 1T′-$MoTe_2$, a KPFM was employed to characterize the surface potential in Supplementary Fig. 3c. The contact potential difference (CPD) between the AFM tip (Pt/Ir coated tips) and the sample is defined as[1, 6]:

$$V_{CPD} = (\Phi_{tip} - \Phi_{sample})/q \quad (1)$$

where Φ_{tip} (5.2-5.6 eV), Φ_{sample}, and q are the work functions of the tip and sample, and the elementary charge, respectively. Bases on the work function of 5.10 eV of Au substrate, the work function of other materials can be calculated according to the following formula:

$$V_{sample1} - V_{Au} = (\Phi_{tip} - \Phi_{sample1})/q - (\Phi_{tip} - \Phi_{Au})/q = (\Phi_{Au} - \Phi_{sample1})/q$$

$$\Phi_{sample1} = \Phi_{Au} - (V_{sample1} - V_{Au}) * q \quad (2)$$

The work function of 1T′-$MoTe_2$ film is extracted as approximately 4.83 eV. Besides, the surface potential of the metallic 1T′-$MoTe_2$ is independent of the film thickness in Supplementary Fig. 3c, which is completely different from semiconducting TMDCs[7].

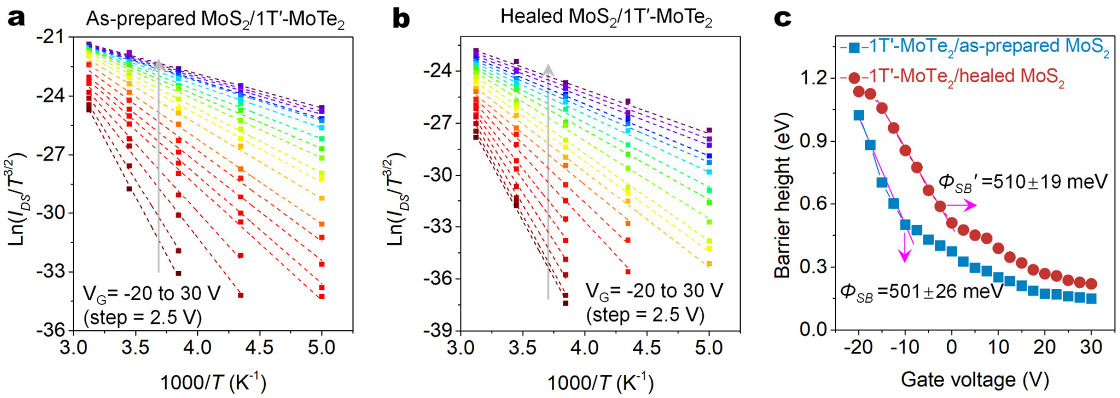

Supplementary Figure 4. Schottky barrier heights of the 1T′-MoTe$_2$/MoS$_2$ Schottky junctions before and after the treatment. a-b, Gate-dependent ln($I_{DS}/T^{3/2}$) versus 1000/T plots in the 1T′-MoTe$_2$/MoS$_2$ Schottky junctions before (**a**) and after (**b**) the SVSH. **c**, Barrier heights of the 1T′-MoTe$_2$/as-prepared MoS$_2$ and 1T′-MoTe$_2$/healed MoS$_2$ Schottky junctions as a function of gate voltage. The Schottky barrier heights are extracted under a flat band gate voltage condition, which is responsible for the start of deviations from the linear behavior.

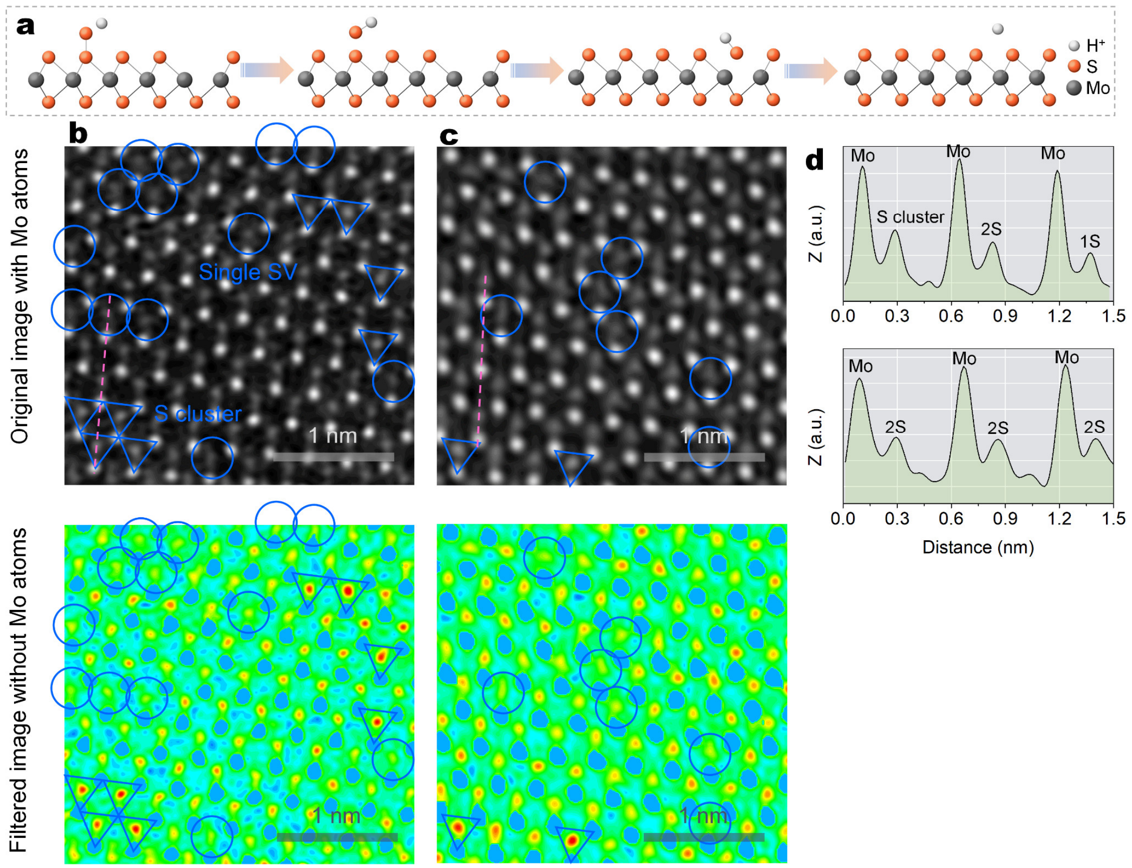

Supplementary Figure 5. STEM images of monolayer MoS_2 before and after the treatment. **a**, Mechanism schematic diagram of the acid-induced SVSH in monolayer MoS_2. **b-c,** Original (top) and filtered (bottom) STEM images of the as-prepared (b) and healed (c) monolayer MoS_2. Blue circles and triangles represent SVs (1S) and sulfur clusters, respectively. **d**, Z-contrast mapping done before (top) and after (bottom) in the areas marked with pink dotted lines in **b** and **c**.

The principle of the SVSH is that the SVs are healed spontaneously by the sulfur adatom clusters on the monolayer MoS_2 surface through an acid-induced hydrogenation process in Supplementary Fig. 5a. To clarify the atomic structure variation, STEM was employed to determine the defect concentration variation of monolayer MoS_2 before and after healing at the atomic scale. As the intensity of STEM images is directly related to the atomic number (Z-contrast), SVs (1S) and sulfur adatom clusters can be visualized and differentiated from the three-fold coordinated two sulfur atoms (Supplementary Fig. 5b and 5c). Besides, to quickly and efficiently distinguish these lattice defects, the interference of Mo atoms was filtered in filtered STEM images without Mo atoms in Supplementary Fig. 5b and S5c bottom. Disordered regions, in which contrast is significantly lower than the nearest six S atom sites, can be

considered as SVs. While disordered regions, which contrast is significantly higher than the nearest six S atom sites, refer to as sulfur clusters. This contrast fluctuation was also confirmed by the extracted Z-value mapping in Supplementary Fig. 5d.

Compared with the as-prepared MoS_2, both the SV and sulfur cluster concentrations of the healed MoS_2 showed a significant decrease in Supplementary Fig. 5b and S5c. Thus, the reduction of SV concentration in healed MoS_2 can be attributed to the acid-induced SVSH effect of the PEDOT:PSS solution[6]. According to a large number of data statistics, the S/Mo ratios in monolayer MoS_2 before and after treatment are ~1.85 and ~1.92, respectively.

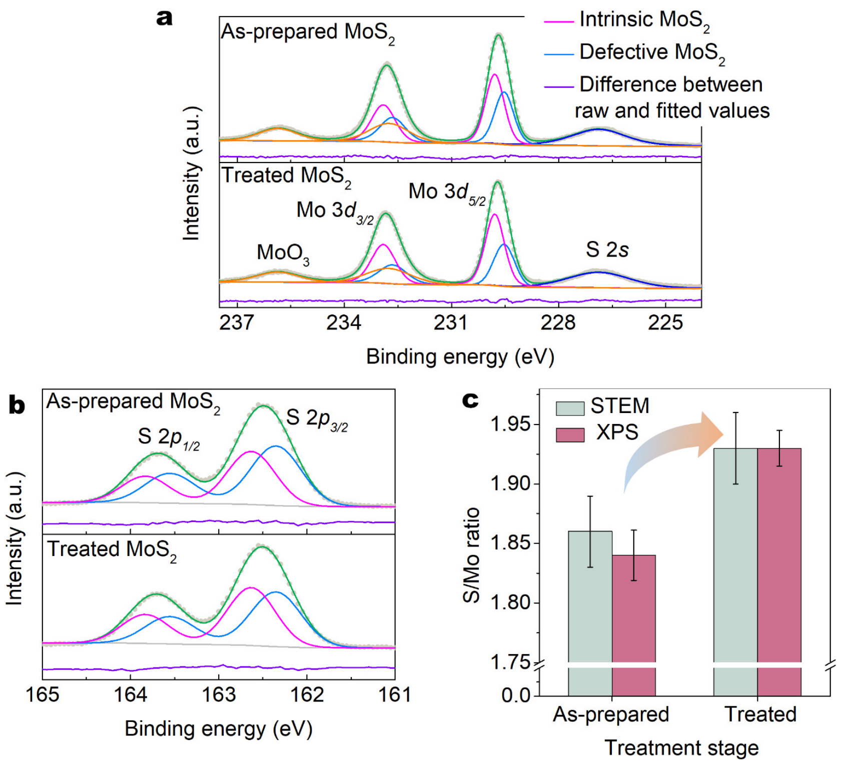

Supplementary Figure 6. XPS spectra of monolayer MoS_2 before and after the treatment. a-b, Mo 3d (a) and S 2p (b) core-level spectra for the as-prepared and treated MoS_2 monolayers on the SiO_2/Si substrates. Chemical contributions from MoO_3, intrinsic MoS_2 (i-MoS_2), and defective MoS_2 (d-MoS_2) are shown. The residual fit lines (purple) between the raw and fitted data are uniform, suggesting our fitting method is credible. **c**, S/Mo ratios extracted from STEM and XPS at different treatment stages.

XPS analyses of the two samples were performed to establish the systematic variations of SVs induced by the acid-induced SVSH (Supplementary Fig. 6a-b). In Mo 3d spectra of the as-prepared monolayer MoS_2, both Mo^{4+} and Mo^{6+} doublets are observed. The Mo^{6+} doublets include the Mo^{6+} $3d_{5/2}$ of 232.74 eV and Mo^{6+} $3d_{3/2}$ of 235.86 eV. The doublet peaks of Mo^{4+} $3d_{5/2}$ and $3d_{3/2}$ can be deconvoluted into two components of intrinsic MoS_2 (i-MoS_2, ~229.79 and ~232.91 eV) and defective MoS_2 (d-MoS_2, ~229.53 and ~232.65 eV). When the doublets of the healed sample are decomposed using the same components of i-MoS_2 and d-MoS_2, the contribution of the defective MoS_2 (d-MoS_2) decreases, whereas the intrinsic MoS_2 (i-MoS_2) component increases. Similarly, the contribution of the i-MoS_2 (~162.63 and ~163.83 eV) in S *2p* doublet peaks of the healed MoS_2 raised, while the component of d-MoS_2 (~162.35 and ~163.55 eV) declined. Similar behaviors were previously observed in SV healed MoS_2 through sulfurization annealing[8]. To quantify the XPS information, we have calculated the XPS peak area ratio of S *2p* to Mo^{4+} *3d* states for the as-prepared and healed MoS_2. The value of the bonded S/Mo ratio was increased from ~1.84 to ~1.93 by the acid-induced SVSH, which is largely consistent with the conclusion based on the STEM analysis in Supplementary Fig. 6c.

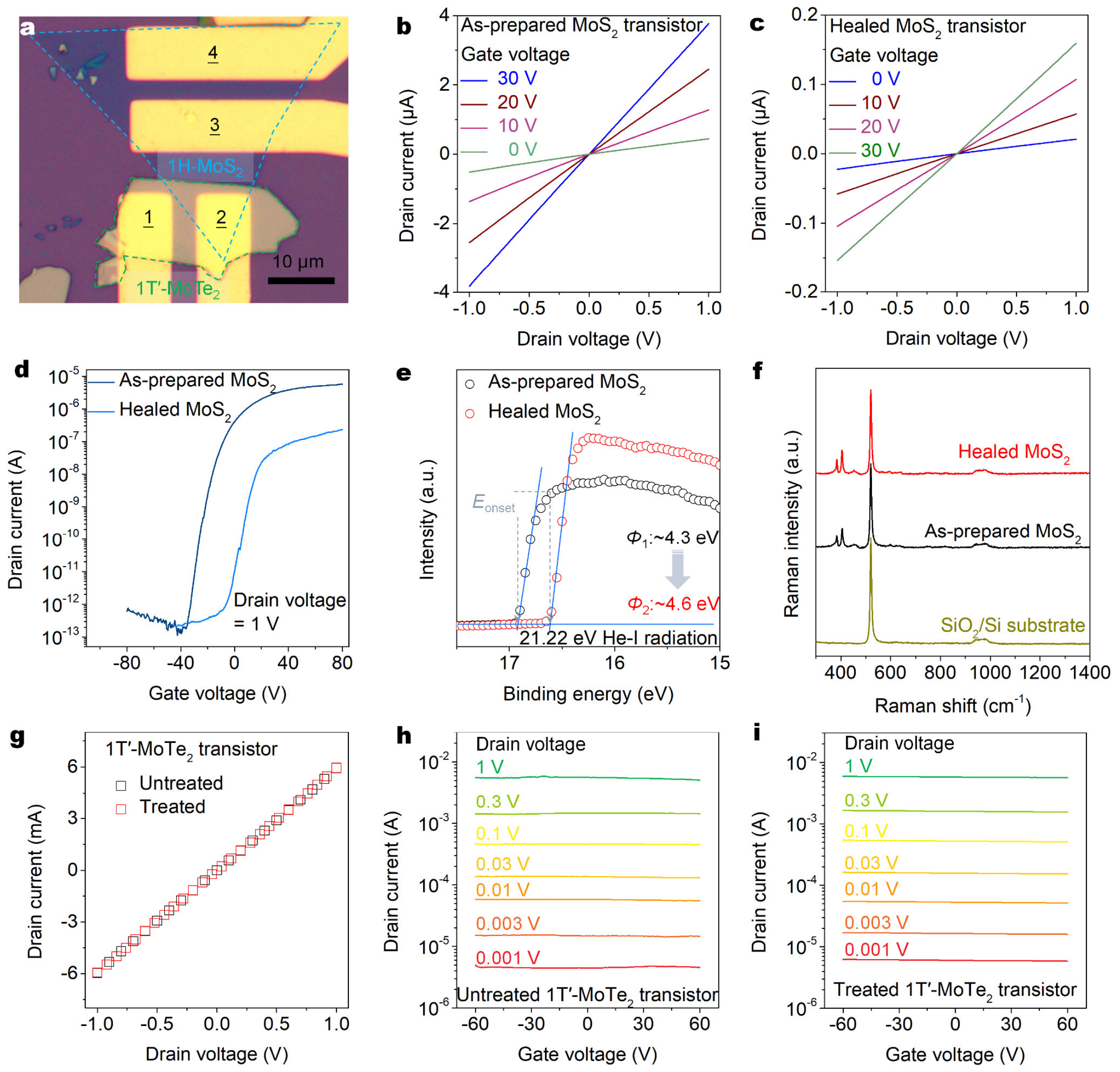

Supplementary Figure 7. Electrical properties of the 1H-MoS_2 and 1T′-$MoTe_2$ transistors before and after the treatment. a, Optical micrograph of a 1T′-$MoTe_2$/MoS_2 Schottky junction. Fig. 3a is the zoom-in of **a**. **b-d**, Output (**b-c**) and transfer (**d**) curves of the 1H-MoS_2 transistors with #3 and # 4 electrodes before and after the SVSH. **e-f**, Secondary-edge spectra of the UPS measurement (**e**) and Raman spectra (**f**) from the as-prepared and healed MoS_2 monolayers. There are no Raman peaks of the PEDOT:PSS in **f** (ref.[6]), such as 1130, 1256, and 1370 cm^{-1}, suggesting that the MoS_2 surface hasn't residual PEDOT:PSS. It should be noted that the Raman spectra were acquired from the monolayer MoS_2 shown in **a.** The Raman peaks of 604, 833, 995, and 1264 cm^{-1} of the PMMA aren't obvious[9], suggesting the PMMA residue remaining from the lift-off process also can be negligible. **g**, Output curves of the 1T′-$MoTe_2$ transistors with #1 and #2 electrodes before and after the SVSH. **h-i,** Transfer curves of the 1T′-$MoTe_2$ transistors at different biased voltages before (**h**) and after (**i**) the SVSH.

While the 1T′-$MoTe_2$/MoS_2 metal-semiconductor was being constructed, the 1T′-$MoTe_2$ and MoS_2 field-effect transistors were also being simultaneously constructed to remove the competing interferences from other electrode contacts in Supplementary Fig. 7a. The linear current-voltage relationships in Supplementary Fig. 7b-7c also show that the Cr electrodes and MoS_2 can remain Ohmic contacts both before and after the SVSH. The removal of SVs makes the threshold voltage of the MoS_2 FET close to zero in Supplementary Fig. 7d, illustrating the electron concentration is significantly lowered. The following formula is used to quantitatively calculate the electron concentration N_{2D} of monolayer MoS_2[10]:

$$N_{2D} = C_i\,(V_G - V_{TH})\,/q \quad (3)$$

where $C_i = 1.15\times10^{-4}$ Fm^{-2} is the gate capacitance of the 300 nm SiO_2 dielectric layer, V_G is the gate voltage, V_{TH} is the threshold voltage, and q is the elementary charge. Combined with the V_{TH} (Supplementary Fig. 7d), we can extract the electron concentrations of the as-prepared and healed monolayer MoS_2 at $V_G = 0$ V are 2.51×10^{12} and 6.46×10^{11} cm^{-2}, respectively. Besides, the on-current at high gate voltage is also reduced ~25 times by the solution treatment, which is consistent with other SV healed MoS_2 through sulfurization annealing and molecular chemisorption and could be explained by a hopping transport model[6, 8, 11, 12].

Besides, the decrease of the background electron concentration leads to the raising of the work function (Φ_S) of ~0.3 eV from ~4.3 to ~4.6 eV measured by UPS in Supplementary Fig. 7e. In the UPS test, the large-scale monolayer MoS_2 films were grown on the SiO_2/p-Si substrates and then transferred onto pure Si substrates. The work function (Φ_S) can be calculated by $\Phi_S = h\nu - E_{onset}$, $h\nu$ is the incident photon energy of 21.22 eV.

For the 1T′-$MoTe_2$ transistor with #1 and #2 electrodes, the source-drain current in the transfer curve reached the ~6 mA at 1 V biased voltage and is completely independent of the gate voltage in Supplementary Fig. 7h-7i, which shows that the 1T′-$MoTe_2$ film exhibits the metallic characteristic rather than semiconducting characteristic. The two figures suggest the SVSH effect hasn't modified the metallic properties of the 1T′-$MoTe_2$.

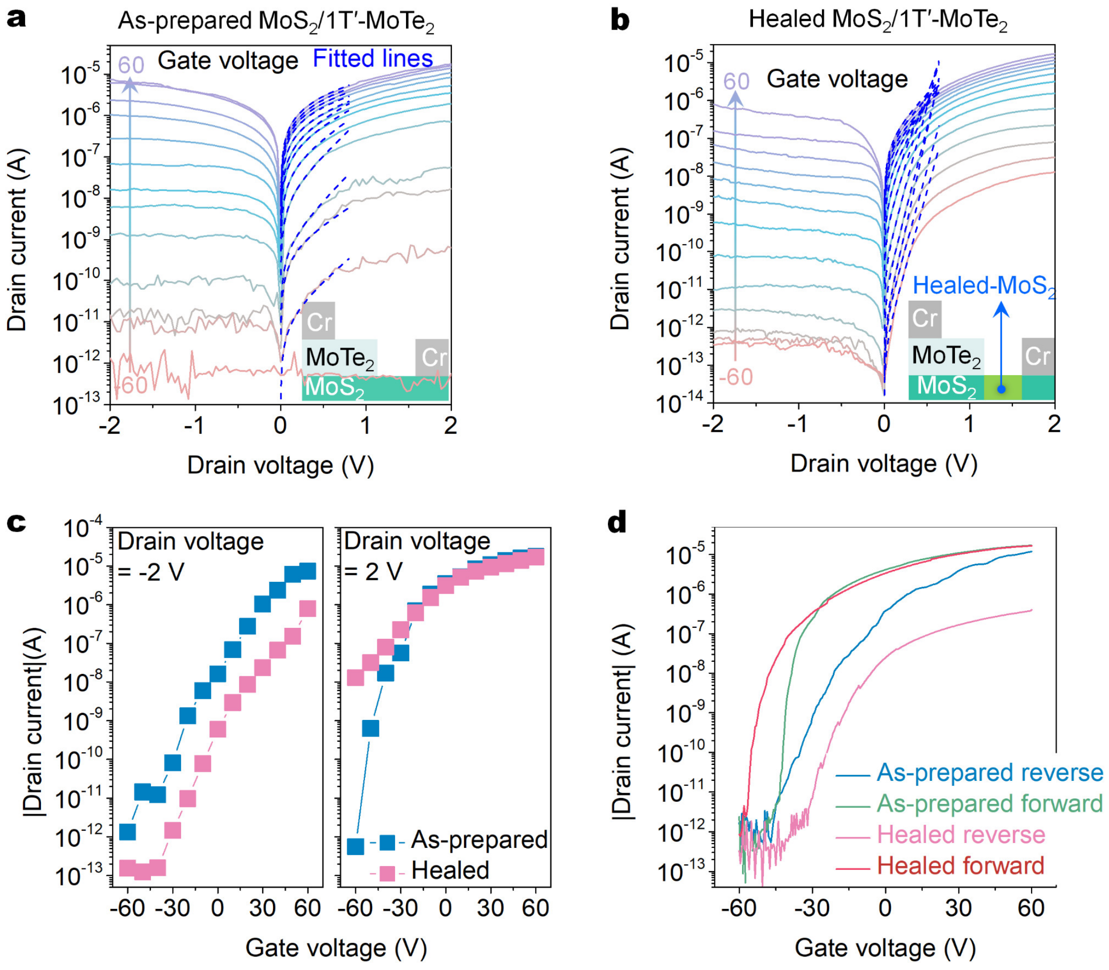

Supplementary Figure 8. Basic electrical characterizations of the 1T′-$MoTe_2$/MoS_2 Schottky junctions. a-b, Gate-dependent output curves in logarithmic scales of the 1T′-$MoTe_2$/MoS_2 Schottky junctions before (**a**) and after (**b**) the SVSH. Insets are corresponding schematic diagrams of the devices before and after the SVSH. **c**, Transfer curves of the 1T′-$MoTe_2$/MoS_2 Schottky junctions before and after the SVSH at forward (+2 V) and reverse (-2 V) biased voltages. **d**, Gate-dependent forward (+2 V) and reverse (-2 V) current extracted from **a** and **b** before and after the SVSH. The diode does not show obvious p-type conductance even at a large gate voltage of -50~-60 V, which may be related to the n-type doping of the defect states within the SiO_2-MoS_2 interface[13].

With the gate voltage sweeping from positive to negative, the 1T′-$MoTe_2$/as-prepared MoS_2 Schottky junction will transform the behavior from Ohmic to rectifying in Supplementary Fig. 8a. However, the 1T′-$MoTe_2$/healed MoS_2 Schottky junction shows obvious rectifying behavior in the full gate voltage regime between -60 and 60 V in

Supplementary Fig. 8b, after the SVs of the CVD-grown MoS_2 healed by the SVSH. The discrepancy of the reverse-biased currents between the 1T′-$MoTe_2$/as-prepared MoS_2 and 1T′-$MoTe_2$/healed MoS_2 Schottky junctions is significantly large in Supplementary Fig. 8c left. Under reverse bias, enlarging the Schottky barrier width can transform the charge injection style from thermionic emission to thermionic field emission (also call thermally assisted tunneling) in Fig. 3g. While the discrepancy of the forward-biased currents is very small in Supplementary Fig. 8c right. The gate-tunable variation trends of both the forward and reverse currents extracted from the output curves are similar to that of the transfer curves in Supplementary Fig. 8d, indicating that the rectifying behaviors are not measured accidentally but reliable. More detailed explanations for the discrepancy can be obtained in Fig. 3f-g.

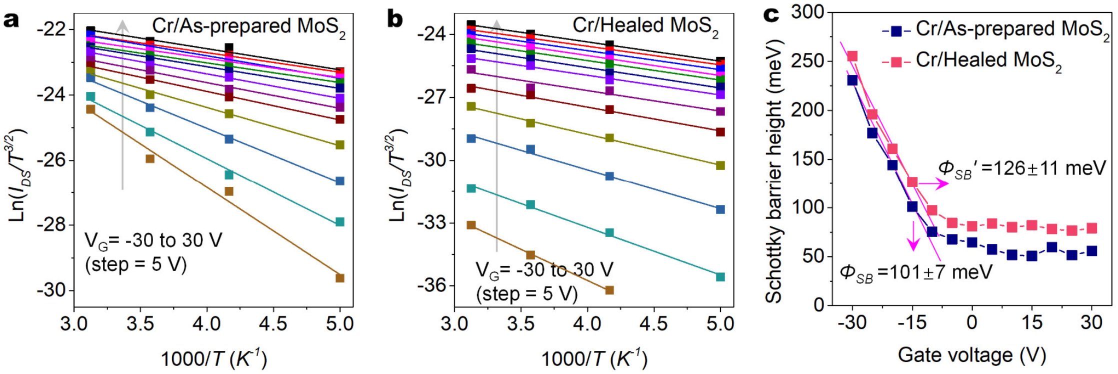

Supplementary Figure 9. Schottky barrier heights of the Cr/MoS_2 contacts before and after the treatment. a-b, Gate-dependent ln($I_{DS}/T^{3/2}$) versus 1000/T plots in the Cr/MoS_2 contacts before (**a**) and after (**b**) the SVSH. **c**, Barrier heights of the Cr/as-grown MoS_2 and Cr/healed MoS_2 contacts as a function of gate voltage. The Schottky barrier heights are extracted under a flat band gate voltage condition, which is responsible for the start of deviations from the linear behavior.

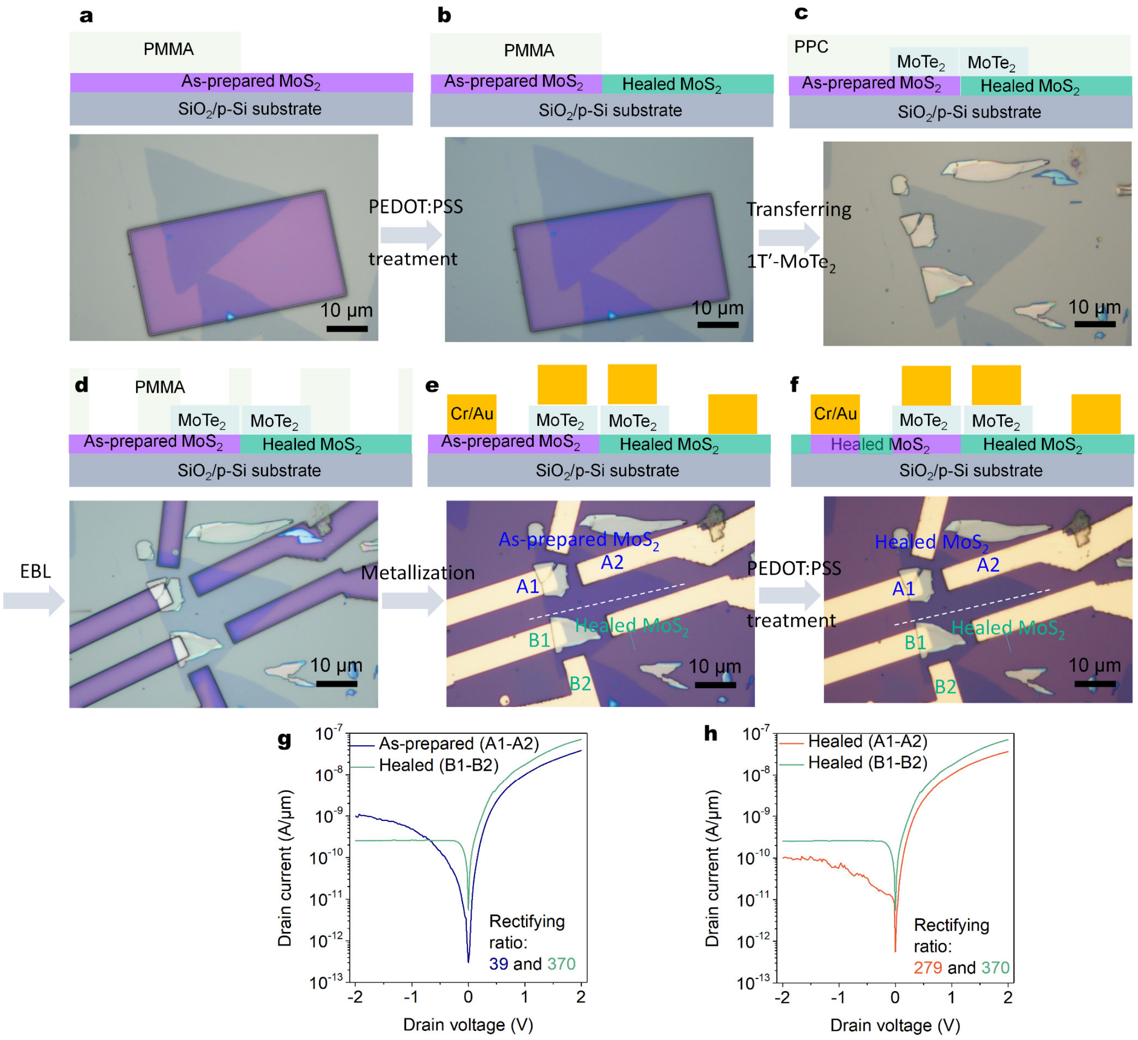

Supplementary Figure 10. Rectifying performance comparisons of the two Schottky junctions with different metal-semiconductor contacts. a-f, Schematic diagrams (top) and optical images (bottom) of each step in the construction process of the two-kind Schottky junctions. **g-h**, Output curves in logarithmic scales of the two-interface Schottky diodes before and after the secondary PEDOT:PSS treatments. All gate voltages are zero. The rectifying performances shown in **g** and **h** refer to the two-interface Schottky diodes in **e** and **f**.

The specific experiment in Supplementary Fig. 10 shows that even if the PEDOT:PSS treatment heals the SVs of the covered MoS_2 of the 1T′-$MoTe_2$/MoS_2 diode, the enhancement behavior of the rectifying performance won't be greatly changed. Since the rectifying ratio of ~39 of the as-prepared (A1-A2) diodes at V_D= ±2 V and V_G= 0 V is smaller than that of ~370 of the healed (B1-B2) diodes in Supplementary Fig. 10g. Furthermore, by the secondary PEDOT:PSS treatment, the rectifying ratio of the as-prepared (A1-A2) diode was increased from ~39 to ~279, which is comparable to that of ~370 of the healed (B1-B2) diode

(Supplementary Fig. 10h).

The possible reason why this contact fluctuation of the covered MoS_2 is independent of the rectifying performance is that, in such Schottky junctions with a large Schottky barrier height of ~0.5 eV, the electron concentration of the covered monolayer MoS_2 won't be affected by the PSS-induced SVSH effect but is mainly determined by the work function of the metal electrode 1T′-$MoTe_2$ (Fig. 3f-g). Since the covered monolayer MoS_2 is very thin and completely in the depletion region of the 1T′-$MoTe_2$/MoS_2 Schottky barrier, the work function of this covered region MoS_2 should be largely equal to the work function of ~4.8 eV of the 1T′-$MoTe_2$ (Supplementary Fig. 3). The work function of ~4.8 eV of the covered region MoS_2 is significantly beyond the adjustment range of the work function from ~4.3 to ~4.6 eV by the PEDOT:PSS treatment (Supplementary Fig. 7e). In other words, after the as-prepared and healed MoS_2 monolayers are contacted to 1T′-$MoTe_2$ flake, their work functions will eventually increase from 4.3 eV and 4.6 eV to 4.8 eV. This means that the work functions of the contacted MoS_2 monolayers won't be affected whether it is healed or not.

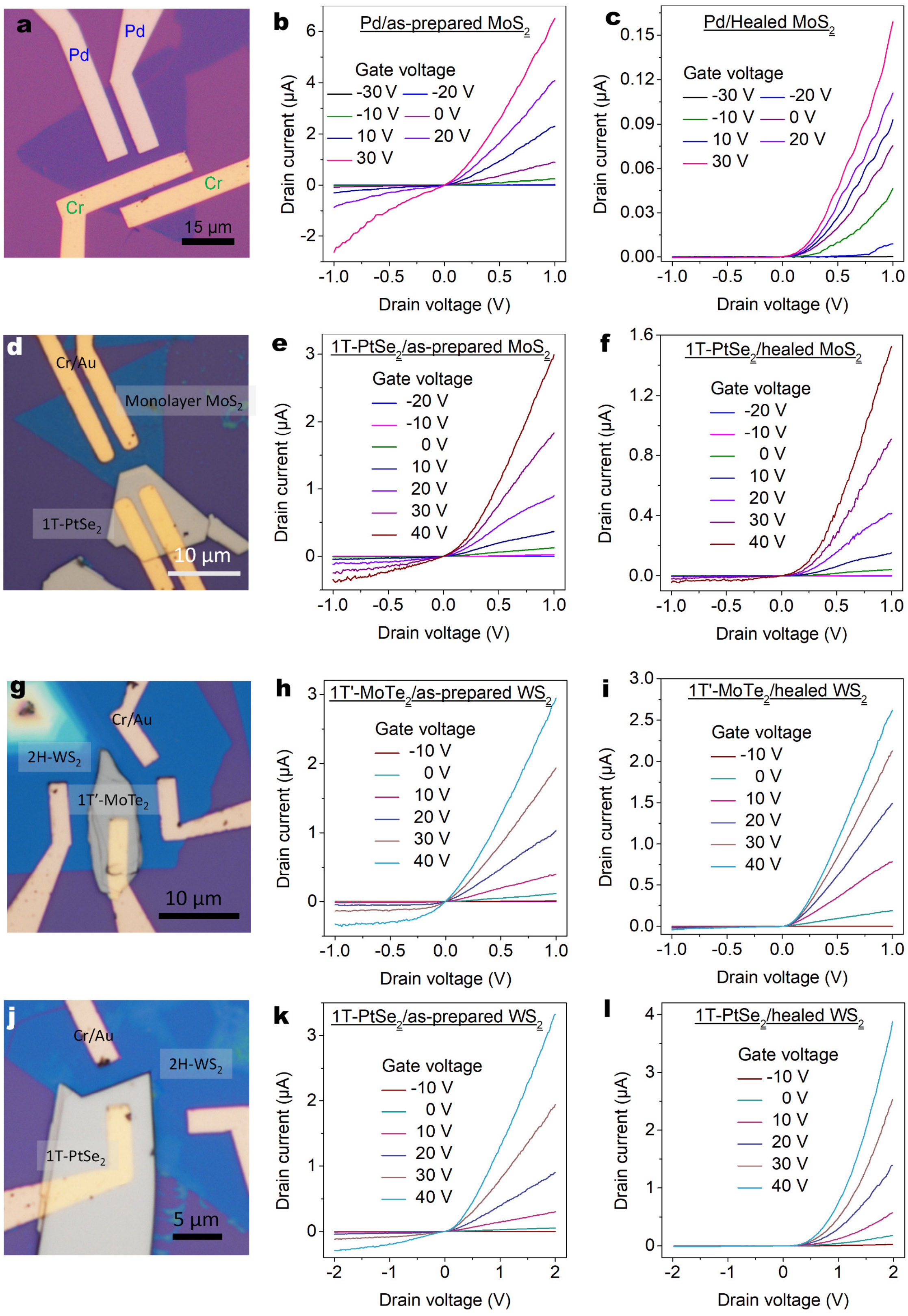

Supplementary Figure 11. Performance comparisons of several Schottky junctions before and after the SVSH. a, **d**, **g**, and **j**, Optical micrographs of the Pd/MoS_2 (**a**), 1T-$PtSe_2$/MoS_2 (**d**),

1T′-$MoTe_2$/WS_2 (**g**), and 1T-$PtSe_2$/WS_2 (**j**) Schottky junction diodes. **b-c**, **e-f**, **h-i**, and **k-l**, Gate-dependent output curves of the four Schottky junction diodes before (**b**, **e**, **h**, and **k**) and after (**c**, **f**, **i**, and **l**) the SVSH.

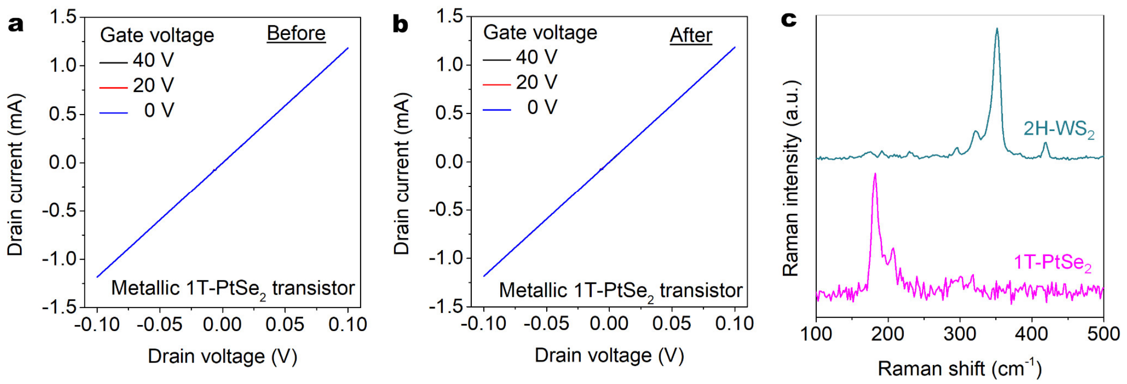

Supplementary Figure 12. Electrical properties of the 1T-$PtSe_2$ electrodes before and after the treatment. a-b, Output curves of the 1T-$PtSe_2$ transistors before (**a**) and after (**b**) the PEDOT:PSS solution treatment. Similar to the 1T′-$MoTe_2$, the metallic behavior of the 1T-$PtSe_2$ is independent of the solution treatment. **c**, Raman spectra of the 2H-WS_2 and 1T-$PtSe_2$ films.

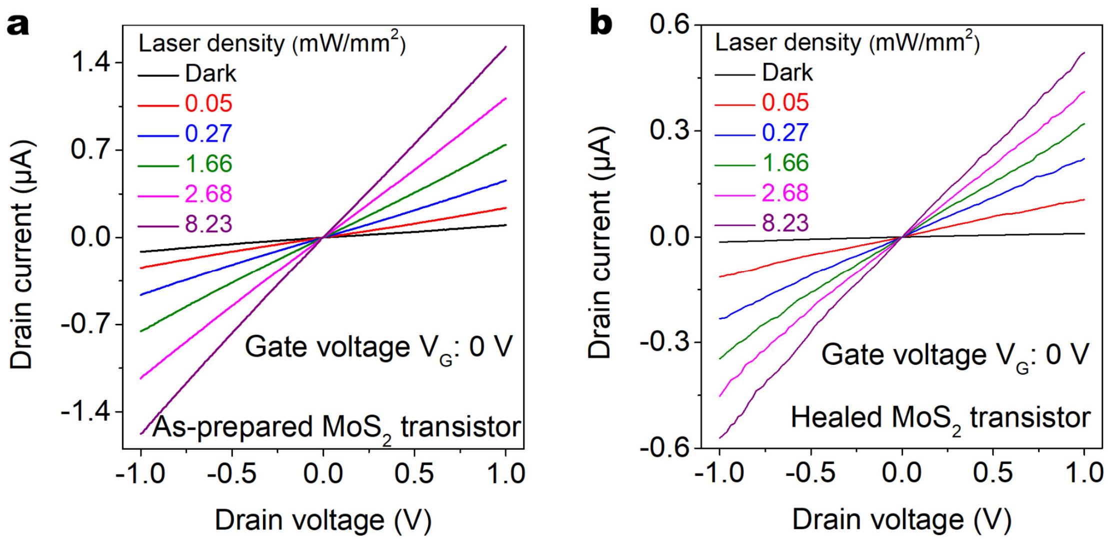

Supplementary Figure 13. Photoresponse characterization of the MoS_2 transistors. a-b, Output curves of the as-prepared (**a**) and healed (**b**) monolayer MoS_2 transistors as a function of the laser power density.

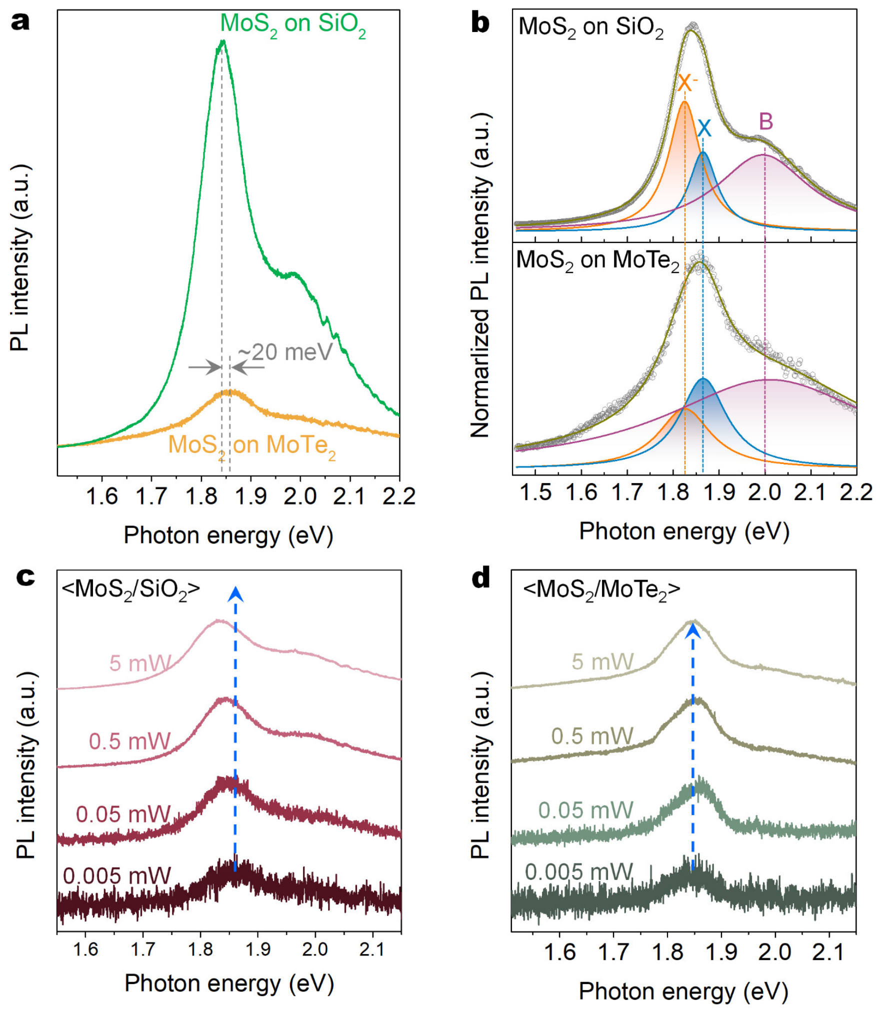

Supplementary Figure 14. PL spectra of the 1T′-$MoTe_2$/MoS_2 Schottky junction interface. a, PL spectra of monolayer MoS_2 under two different substrates of insulator SiO_2 and metallic 1T′-$MoTe_2$. **b**, Comparison of the deconvoluted PL spectrum features in **a**. The experimental results are reproduced by the sum (yellow) of three peaks (trion X^-, orange; exciton X, blue; exciton B, purple) with Lorentzian functions. **c-d**, Laser intensity-dependent PL spectra of the monolayer MoS_2 on insulating SiO_2 substrate (**c**) and metallic $MoTe_2$ film (**d**).

In general, the thickness of 0.85 nm of monolayer MoS_2 is much less than the depletion region (>2.9 nm) width of the 1T′-$MoTe_2$/MoS_2 Schottky junctions[14]. Next, the PL spectrum was also characterized to reconfirm the decrease in the electron concentration of MoS_2 in the overlapped depletion region. Compared to the monolayer MoS_2 supported on the SiO_2 insulating substrate, the PL spectrum intensity and peak position of the overlapped region is substantially reduced and blue-shifted of ~20 meV in Supplementary Fig. 14a.

This blue-shift in PL peak position is mainly due to the obvious difference in electron concentration between MoS_2 in the depletion region and MoS_2 on the insulating substrate. Whether supported by SiO_2 or $MoTe_2$, the PL spectra of monolayer MoS_2 can be broken down into B excitons, intrinsic excitons (X), and trions (X^-) by peak fitting in Supplementary Fig. 14b. A trion in n-type monolayer MoS_2 is mainly composed of two electrons and one hole, and its component is largely positive with the degree of the electron concentration[10, 11]. Different from the MoS_2 supported by SiO_2, the components of intrinsic excitons (X) and trions (X^-) of the PL spectrum in MoS_2 on $MoTe_2$ are dominant and little, suggesting that electron concentration in monolayer MoS_2 on $MoTe_2$ is lower than that of the MoS_2 on SiO_2.

It has been previously reported that decreasing the electron concentration of MoS_2 on the insulating substrate by gate doping can blue-shift the peak position by 20-30 meV but enhance the total PL spectral intensity[15]. Here's the odd thing that the total PL spectral intensity of the MoS_2 on $MoTe_2$ is lower than that of the MoS_2 on SiO_2, which can be attributed to the efficient photocarrier separation driven by the Schottky built-in electrical field. With the increase of the laser intensity, The PL peak position of monolayer MoS_2 on SiO_2 is continuously red-shifted in Supplementary Fig. 14c, while the PL spectrum in the Schottky interfaces is indeed substantially unchanged in Supplementary Fig. 14d. This can be attributed to that with the increase of the laser intensity, more electrons are excited from the valence band maximum to the conduction band minimum but cannot be effectively separated, thereby forming a large number of trions to increase the relative component of trions in MoS_2 on SiO_2. For the Schottky interfaces, the electrons excited to the conduction band minimum are quickly separated and hardly used to form trions and alter the peak position[1]. Most of the unseparated photocarriers in MoS_2 on $MoTe_2$ are characterized by intrinsic excitons rather than trions, which are limited by insufficient electron concentration to form trions.

a

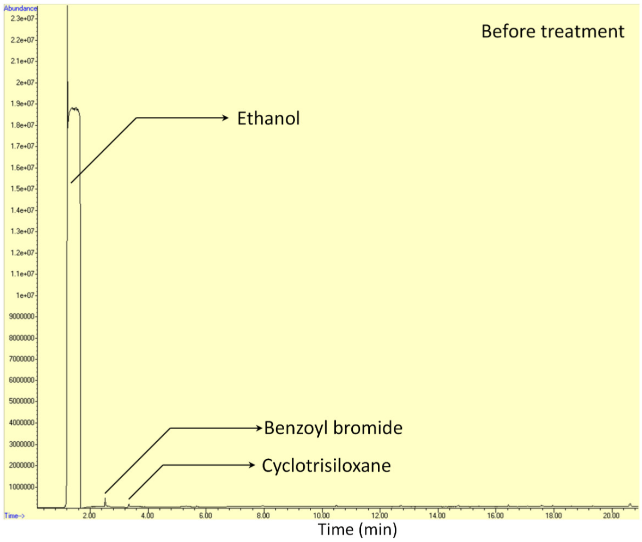

b

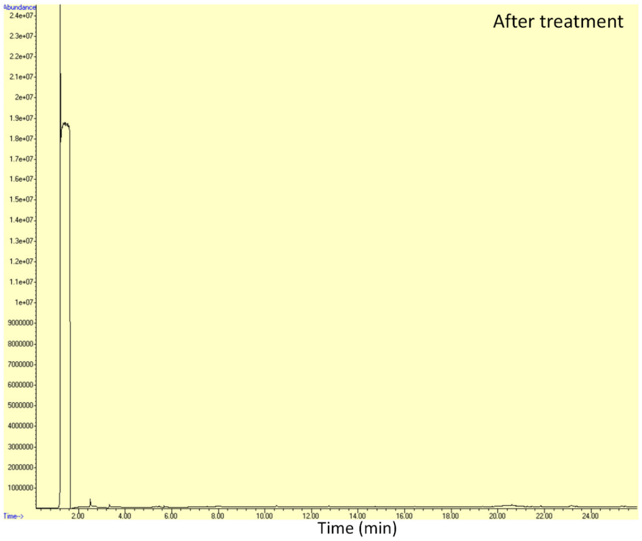

Supplementary Figure 15. Removal of the residual PEDOT:PSS in the Schottky diodes. a-b, GC-MS spectra of the 1T′-$MoTe_2$/MoS_2 Schottky diodes before (a) and after (b) the PEDOT:PSS treatment.

GC-MS is a very effective way to characterize trace residues of organic matter. As shown in Supplementary Figs. 15a-b, the PEDOT:PSS processed samples did not introduce additional residues, indicating that a large amount of DI water (>200 + >200 mL) could completely remove PEDOT:PSS. Although the spectra have detected three substances (ethanol, cyclotrisiloxane, and benzoyl bromide), none of them were related to the PEDOT:PSS residues in Figs. S15a-b. The absolute ethanol was used as the solvent of the GC-MS sample to fully extract possible residues, the cyclotrisiloxane was released by the silica gel pipe of the GC-MS, and benzoyl bromide should be an impurity in absolute ethanol. Combined with the Raman spectra (Supplementary Fig. 7f), we can conclude that the enhancement effect of rectifying behavior of the PEDOT:PSS treatment wasn't derived from the residue of PEDOT:PSS.

Supplementary Table 1. Performance survey of 2D semiconductor-based Schottky diodes.

Materials	Configuration	Rectifying ratio/Biased voltage (V)	Ideality factor	I_{SC}(nA)/ V_{OC}(V)	Ref.
Monolayer MoS_2	1T′-$MoTe_2$ electrode	$5.1\times10^5/\pm2$	1.6	22/0.19	This work
Monolayer MoS_2	1T′-MoS_2 electrode	$3\times10^3/\pm10$	N/A	N/A	Ref. [16]
	[+]G/MoS_2 Schottky junction	$2\times10^4/\pm3$	N/A	No PV[×]	Ref. [17]
	Epitaxial growth G electrode	$10^6/\pm10$<br>$3\times10^4/\pm2$	1.5	No PV	Ref. [18]
	Evaporated Ti/Pt electrodes	104/±10	N/A	N/A	Ref. [19]
	Transferred Ag/Pt electrodes	$4\times10^4/\pm1.5$	N/A	2.4/1.02	Ref. [13]
	Molecule-doping and Pt/Ir tip	$5\times10^4/\pm1$	N/A	No PV	Ref. [20]
Multilayer MoS_2	Transferred Ag/Pt electrodes	$10^8/\pm1$	1.09	11/0.76	Ref. [13]
	Evaporated Ag/Pt electrodes	N/A	1.8	1/0.3	
	Evaporated Au/Pd electrodes	4.5/±0.15	N/A	1.8/0.1	Ref. [21]
	Evaporated Ti/Pd electrodes	~100/±0.5	1.2	N/A	Ref. [22]
	Evaporated Ti/transferred G electrodes	2/±5	N/A	N/A	Ref. [23]
	Evaporated Cr/transferred G electrodes	~40/±1	1.1	N/A	Ref. [24]
	Vertical Au/Pd electrodes	~5/±0.5	N/A	200/0.08	Ref. [25]
	Evaporated Pd/1T′-MoS_2 electrodes	~30/±0.5	N/A	N/A	Ref. [26]
	Oxidized Ti contacts	$\sim3\times10^4/\pm40$	48	N/A	Ref. [27]
	Au/MoS_2/NbS_2	$10^5/\pm3$	4.0	N/A	Ref. [28]
	G/MoS_2/NbS_2	$10^4/\pm3$	1.6	N/A	
Multilayer WS_2	Vertical G electrode with BN buffer layer	$4\times10^5/\pm1$	1.2	N/A	Ref. [29]
Multilayer BP*	Evaporated Au/Al electrodes	$1.5\times10^3/\pm1$	N/A	1/0.05	Ref. [30]
Multilayer WSe_2	Gate-modulated WSe_2/G contact	$1.5\times10^4/\pm5$	1.1	N/A	Ref. [31]
	Vertical Gd/Pt electrodes	$10^3/\pm0.3$	1.16	N/A	Ref. [32]
	Vertical Ag/G electrodes	$5\times10^2/\pm0.3$	N/A	N/A	Ref. [33]
Multilayer lnSe	Evaporated Au/Ag electrodes	$2\times10^6/\pm2$	1.097	200/0.4	Ref. [34]
	Au and G electrodes	68/±1	2.2	0.14/0.1	Ref. [35]
Multilayer ln_2Se_3	Metallic β-In_2Se_3 electrodes	$4\times10^2/\pm1$	N/A	N/A	Ref. [36]

[#]N/A indicates "not applicable". [+]G represents graphene. [×]PV represents photovoltaic. *BP represents black phosphorus.

Supplementary References

1. Liu B, *et al.* Strain-engineered van der Waals interfaces of mixed-dimensional heterostructure arrays. *ACS Nano* **13,** 9057-9066 (2019).
2. Li X-L, Han W-P, Wu J-B, Qiao X-F, Zhang J, Tan P-H. Layer-number dependent optical properties of 2D materials and their application for thickness determination. *Adv. Funct. Mater.* **27,** 1604468 (2017).
3. Zhang X, *et al.* Self-healing originated van der Waals homojunctions with strong interlayer coupling for high-performance photodiodes. *ACS Nano* **13,** 3280-3291 (2019).
4. Luong DH, *et al.* Tunneling photocurrent assisted by interlayer excitons in staggered van der waals hetero-bilayers. *Adv. Mater.* **29,** 1701512 (2017).
5. Chakraborty B, Bera A, Muthu DVS, Bhowmick S, Waghmare UV, Sood AK. Symmetry-dependent phonon renormalization in monolayer MoS_2 transistor. *Phys. Rev. B* **85,** 161403 (2012).
6. Zhang X, *et al.* Poly(4-styrenesulfonate)-induced sulfur vacancy self-healing strategy for monolayer MoS_2 homojunction photodiode. *Nat. Commun.* **8,** 15881 (2017).
7. Duong NT, Lee J, Bang S, Park C, Lim SC, Jeong MS. Modulating the functions of MoS_2/$MoTe_2$ van der Waals heterostructure via thickness variation. *ACS Nano* **13,** 4478-4485 (2019).
8. Kim IS, *et al.* Influence of stoichiometry on the optical and electrical properties of chemical vapor deposition derived MoS_2. *ACS Nano* **8,** 10551-10558 (2014).
9. Thakur VK, Vennerberg D, Madbouly SA, Kessler MR. Bio-inspired green surface functionalization of PMMA for multifunctional capacitors. *RSC Adv.* **4,** 6677 (2014).
10. Gao L, *et al.* Defect-engineered atomically thin MoS_2 homogeneous electronics for logic inverters. *Adv. Mater.* **32,** e1906646 (2020).
11. Amani M, *et al.* Near-unity photoluminescence quantum yield in MoS_2. *Science* **350,** 1065-1068 (2015).
12. Cho K, *et al.* Electrical and optical characterization of MoS_2 with sulfur vacancy passivation by treatment with alkanethiol molecules. *ACS Nano* **9,** 8044-8053 (2015).
13. Liu Y, *et al.* Approaching the Schottky-Mott limit in van der Waals metal-semiconductor junctions. *Nature* **557,** 696-700 (2018).
14. Schulman DS, Arnold AJ, Das S. Contact engineering for 2D materials and devices. *Chem. Soc. Rev.* **47,** 3037-3058 (2018).
15. Mak KF, *et al.* Tightly bound trions in monolayer MoS_2. *Nat. Mater.* **12,** 207-211 (2013).
16. Sun L, *et al.* Layer-dependent chemically induced phase transition of two-dimensional MoS_2. *Nano Lett.* **18,** 3435-3440 (2018).
17. Huang H, *et al.* High-performance two-dimensional Schottky diodes utilizing chemical vapour deposition-grown graphene-MoS_2 heterojunctions. *ACS Appl. Mater. Interfaces* **10,** 37258-37266 (2018).
18. Deng W, *et al.* High detectivity from a lateral graphene-MoS_2 Schottky photodetector grown by chemical vapor deposition. *Adv. Electron. Mater.* **4,** 1800069 (2018).
19. Yoon HS, *et al.* Layer dependence and gas molecule absorption property in MoS_2 Schottky diode with asymmetric metal contacts. *Sci. Rep.* **5,** 10440 (2015).
20. Margapoti E, *et al.* A 2D semiconductor-self-assembled monolayer photoswitchable diode. *Adv. Mater.* **27,** 1426-1431 (2015).
21. Fontana M, *et al.* Electron-hole transport and photovoltaic effect in gated MoS_2 Schottky junctions. *Sci. Rep.* **3,** 1634 (2013).
22. Kim JS, *et al.* Multifunctional Schottky-diode circuit comprising palladium/molybdenum disulfide nanosheet. *Small* **10,** 4845-4850 (2014).
23. Lee YT, *et al.* Graphene versus ohmic metal as source-drain electrode for MoS_2 nanosheet transistor channel.

Small **10,** 2356-2361 (2014).

24. Tian H, *et al.* Novel field-effect Schottky barrier transistors based on graphene-MoS_2 heterojunctions. *Sci. Rep.* **4,** 5951 (2014).
25. Li Z, Chen J, Dhall R, Cronin SB. Highly efficient, high speed vertical photodiodes based on few-layer MoS_2. *2D Mater.* **4,** 015004 (2016).
26. Zhang X, *et al.* Two-dimensional MoS_2-enabled flexible rectenna for Wi-Fi-band wireless energy harvesting. *Nature* **566,** 368-372 (2019).
27. Di Bartolomeo A, *et al.* Asymmetric Schottky contacts in bilayer MoS_2 field effect transistors. *Adv. Funct. Mater.* **28,** 1800657 (2018).
28. Shin HG, *et al.* Vertical and in-plane current devices using NbS_2/n-MoS_2 van der Waals Schottky junction and graphene contact. *Nano Lett.* **18,** 1937-1945 (2018).
29. Chu D, Lee YH, Kim EK. Selective control of electron and hole tunneling in 2D assembly. *Sci. Adv.* **3,** e1602726 (2017).
30. Miao J, Zhang S, Cai L, Wang C. Black phosphorus Schottky diodes: channel length scaling and application as photodetectors. *Adv. Electron. Mater.* **2,** 1500346 (2016).
31. LaGasse SW, Dhakras P, Watanabe K, Taniguchi T, Lee JU. Gate-tunable graphene-WSe_2 heterojunctions at the Schottky-Mott limit. *Adv. Mater.* **31,** e1901392 (2019).
32. Nazir G, *et al.* Ultimate limit in size and performance of WSe_2 vertical diodes. *Nat. Commun.* **9,** 5371 (2018).
33. Huh W, *et al.* Synaptic arristor based on phase-engineered 2D heterostructures. *Adv. Mater.* **30,** e1801447 (2018).
34. Hu S, *et al.* Au-InSe van der Waals Schottky junctions with ultralow reverse current and high photosensitivity. *Nanoscale* **12,** 4094-4100 (2020).
35. Zhao Q, Jie W, Wang T, Castellanos-Gomez A, Frisenda R. InSe Schottky diodes based on van der Waals contacts. *Adv. Function. Mater.* **30,** 2001307 (2020).
36. Wang Q, *et al.* Phase-defined van der Waals Schottky junctions with significantly enhanced thermoelectric properties. *J. Phys. Chem. Lett.* **8,** 2887-2894 (2017).
